# Operational response simulation tool for epidemics within refugee and IDP settlements: A scenario-based case study of the Cox’s Bazar settlement

**DOI:** 10.1371/journal.pcbi.1009360

**Published:** 2021-10-28

**Authors:** Joseph Aylett-Bullock, Carolina Cuesta-Lazaro, Arnau Quera-Bofarull, Anjali Katta, Katherine Hoffmann Pham, Benjamin Hoover, Hendrik Strobelt, Rebeca Moreno Jimenez, Aidan Sedgewick, Egmond Samir Evers, David Kennedy, Sandra Harlass, Allen Gidraf Kahindo Maina, Ahmad Hussien, Miguel Luengo-Oroz

**Affiliations:** 1 United Nations Global Pulse, New York, New York, United States of America; 2 Institute for Data Science, Durham University, Durham, United Kingdom; 3 New York University Stern School of Business, New York, New York, United States of America; 4 MIT-IBM Watson AI Lab, Cambridge, Massachusetts, United States of America; 5 UNHCR Innovation, Geneva, Switzerland; 6 WHO Emergency Sub-Office, Cox’s Bazar, Bangladesh; 7 UK Public Health Rapid Support Team, Public Health England/London School of Hygiene and Tropical Medicine, London, United Kingdom; 8 UNHCR Public Health Unit, Geneva, Switzerland; 9 UNHCR Public Health Unit, Cox’s Bazar, Bangladesh; 10 UNHCR Information Management Unit, Cox’s Bazar, Bangladesh; Institute for Disease Modeling, UNITED STATES

## Abstract

The spread of infectious diseases such as COVID-19 presents many challenges to healthcare systems and infrastructures across the world, exacerbating inequalities and leaving the world’s most vulnerable populations most affected. Given their density and available infrastructure, refugee and internally displaced person (IDP) settlements can be particularly susceptible to disease spread. In this paper we present an agent-based modeling approach to simulating the spread of disease in refugee and IDP settlements under various non-pharmaceutical intervention strategies. The model, based on the June open-source framework, is informed by data on geography, demographics, comorbidities, physical infrastructure and other parameters obtained from real-world observations and previous literature. The development and testing of this approach focuses on the Cox’s Bazar refugee settlement in Bangladesh, although our model is designed to be generalizable to other informal settings. Our findings suggest the encouraging self-isolation at home of mild to severe symptomatic patients, as opposed to the isolation of all positive cases in purpose-built isolation and treatment centers, does not increase the risk of secondary infection meaning the centers can be used to provide hospital support to the most intense cases of COVID-19. Secondly we find that mask wearing in all indoor communal areas can be effective at dampening viral spread, even with low mask efficacy and compliance rates. Finally, we model the effects of reopening learning centers in the settlement under various mitigation strategies. For example, a combination of mask wearing in the classroom, halving attendance regularity to enable physical distancing, and better ventilation can almost completely mitigate the increased risk of infection which keeping the learning centers open may cause. These modeling efforts are being incorporated into decision making processes to inform future planning, and further exercises should be carried out in similar geographies to help protect those most vulnerable.

## Author summary

The spread of infectious diseases presents many challenges to healthcare systems and infrastructures across the world. Given their density and available infrastructure, refugee and internally displaced person (IDP) settlements can be particularly susceptible to the dangers of disease spread.

This study seeks to understand how COVID-19 spreads in settlements, focusing on the Cox’s Bazar refugee settlement in Bangladesh. Our model simulates the movements and interactions of each individual in the settlement, incorporating information about family structures and demographic attributes, to understand how COVID-19 might spread under various intervention strategies.

Our analysis suggests that mask wearing in indoor locations can have a significant effect on disease spread, even when wearing reusable cotton masks, which the people in the settlement can make themselves. We also look at different ways to treat individuals who only have milder symptoms and don’t yet require hospitalisation, as well as various scenarios which might allow for the safe reopening of schools in the settlement.

With almost 80 million forcibly displaced people in the world, we hope that this work will inspire more modeling groups to focus on these vulnerable populations, which have been traditionally under-served by such efforts, to ensure no one is left behind.

## 1 Introduction

The spread of COVID-19 across the world presents many challenges to healthcare systems and infrastructures, exacerbating inequalities and leaving the world’s most vulnerable populations most affected. Refugee and internally displaced persons (IDPs) settlements, especially those which have been rapidly created in response to sudden crises, often suffer from overcrowding and insufficient sanitation facilities. Given these conditions, disease spread in settlements has previously been shown to be rapid [1]. The COVID-19 pandemic presents significant threats to people living in these settlements, and the provision of detailed information on potential mitigation strategies is of vital importance. In this paper, we present a simulation tool to support decision making and advocacy by simulating the potential effectiveness of operational interventions in refugee and IDP settlements.

Specifically, we take an agent-based modeling (ABM) approach to understand the impact of public health interventions on limiting disease spread in settlements. Operational interventions (particularly non-pharmaceutical interventions) can take a variety of forms, from alternative care delivery mechanisms to behavioral interventions such as physical distancing. In settlements, some of the most frequent interventions for mitigating disease spread are not feasible due to the complex environments and difficult conditions in which Persons of Concern (PoCs) to the UN Refugee Agency (UNHCR) live [2]. For example, a lack of Personal Protective Equipment (PPE) such as surgical masks could make well-established measures such as compulsory mask wearing challenging to implement. To overcome these difficulties, new operational interventions must also be devised and evaluated. By simulating the possible effects of such measures, we hope to provide teams on the ground with reliable insights for data-driven decision making and situational planning.

The development and testing of this approach focuses on the Cox’s Bazar refugee settlement in Bangladesh, which reported its first COVID-19 case in mid-May 2020 [3]. In particular, we model the interactions between residents of the Kutupalong-Balukhali Expansion Site. With over 900,000 Rohingya PoCs, Cox’s Bazar contains one of the largest refugee settlements in the world [4]. Its inhabitants are primarily Rohingya people, a stateless Muslim minority who have fled targeted violence, discrimination, and human rights violations in Myanmar [5]. A number of risk factors make the settlement vulnerable to epidemic outbreaks, including: high rates of global acute malnutrition and other comorbidities such as respiratory illnesses, which could lead to lower general immunity among camp residents [6]; high population density and communal facilities, which increase the risk of person-to-person transmission; and limited access to sources of information such as the internet as well as low levels of literacy, which make public health campaigns challenging.

Given these risks, UNHCR and World Health Organization (WHO) teams responded rapidly to the COVID-19 pandemic, initiating preventive activities two months before the first case was confirmed [7–9]. Due to limitations in testing and case reporting in the settlement setting, we take a scenario-based approach focused on simulating the relative efficacies of potential interventions, as opposed to attempting to predict highly accurate numbers for infections, hospitalizations and fatalities. As a result of this design choice, detailed COVID-19 case data is not required for our modeling; instead, we rely on a set of clearly recorded assumptions on interaction and transmission probabilities which can be varied in sensitivity analyses.

Approaches to modeling infectious diseases span a broad range of techniques. Some of the most common methodologies are differential equation-based compartmental models [10]. These approaches are useful for gaining high-level insights based on aggregate data, but the level of granularity offered by these models can be limited. Indeed, in the case of refugee and IDP settlements, continuous reporting by UN entities and NGOs ensures that regular demographic and needs-based data is collected in a consistent manner. Agent-based models are often chosen due to their ability to capture geographic and demographic heterogeneity within a population, as well as differences in behavioral patterns including group-level dynamics and social mixing down to the individual level [11, 12]. This level of granularity allows for the precise simulation of many possible operational interventions, and is becoming increasingly accessible given recent improvements in data collection and computational power. Several studies have used ABMs for modeling infectious diseases and health-related policy interventions in low-resource settings (e.g. [13, 14]). Our work is similar to prior models of cholera spread which incorporate detailed information such as the geographic structure of settlements and the movement of agents to undertake routine daily activities [15, 16].

Recently, a number of models have been developed to simulate the spread of COVID-19 in refugee and IDP settlements specifically. For example, Truelove *et al*. [17] present a compartmental modeling approach simulating the spread of COVID-19 in the Kutupalong-Balukhali Expansion Site of the Cox’s Bazar settlement with a focus on predicting infections, hospitalizations, and mortality based on different transmission scenarios defined by their reproduction number. An agent-based approach has also been used model the spread of the disease and possible interventions in Greece’s Moria camp [18].

The core strengths of our approach are that (i) we present a generalizable framework for simulating epidemics in complex refugee and IDP settings that takes into account detailed data on geography, population structure, behavior, facilities and potential mixing points; (ii) we implement operational interventions as changes in the parameters defining movement patterns, social behaviors or contact intensity, which makes it possible to evaluate a wide range of policy options—including geographically heterogeneous interventions—without fundamentally altering the model structure; (iii) we model detailed health trajectories and account for the impacts of different comorbidities; and (iv) we propose a visual analytics framework that allows us to distill insights from our simulations for public health experts and decision makers. This paper will focus on presenting the first three of these in detail, including their implementation in the case of the Cox’s Bazar settlement, while the visual analytics framework will be presented and discussed in generality.

## 2 Methods

The structure and functionality of our model have been adapted from June [19], a generalizable ABM framework for modeling the movement and interactions of people at the individual level, which was first used to model the spread of COVID-19 in England. Our methodology has been designed to apply not only to the current COVID-19 situation, but also to generalize to situation planning in future disease outbreaks in similar geographies. Our modeling process consists of four stages: i) building a ‘digital twin’ of the community of interest; ii) understanding and simulating the possible movements and interactions of the community’s residents; iii) implementing operational interventions to simulate their effects on the spread of disease; and iv) communicating findings to decision makers and experts in the field. This final step is equally as important as the others since if results cannot be effectively communicated, then valuable insights from the model will not be useful.

### 2.1 Digital twin

The first stage of our modeling process requires building a ‘digital twin’ of the settlement. This consists of defining the geographical structure of the model, building the virtual population and assigning them demographic attributes, and constructing locations where individuals can interact with each other. The digital twin forms the basis for the environment in which the simulation can be run. In this section we describe the model’s construction and provide an overview of our data sources and algorithmic choices. For additional details on the underlying architecture we refer to the original framework publication [19] and to S1 Appendix.

#### 2.1.1 Geography

**Fig 1:**
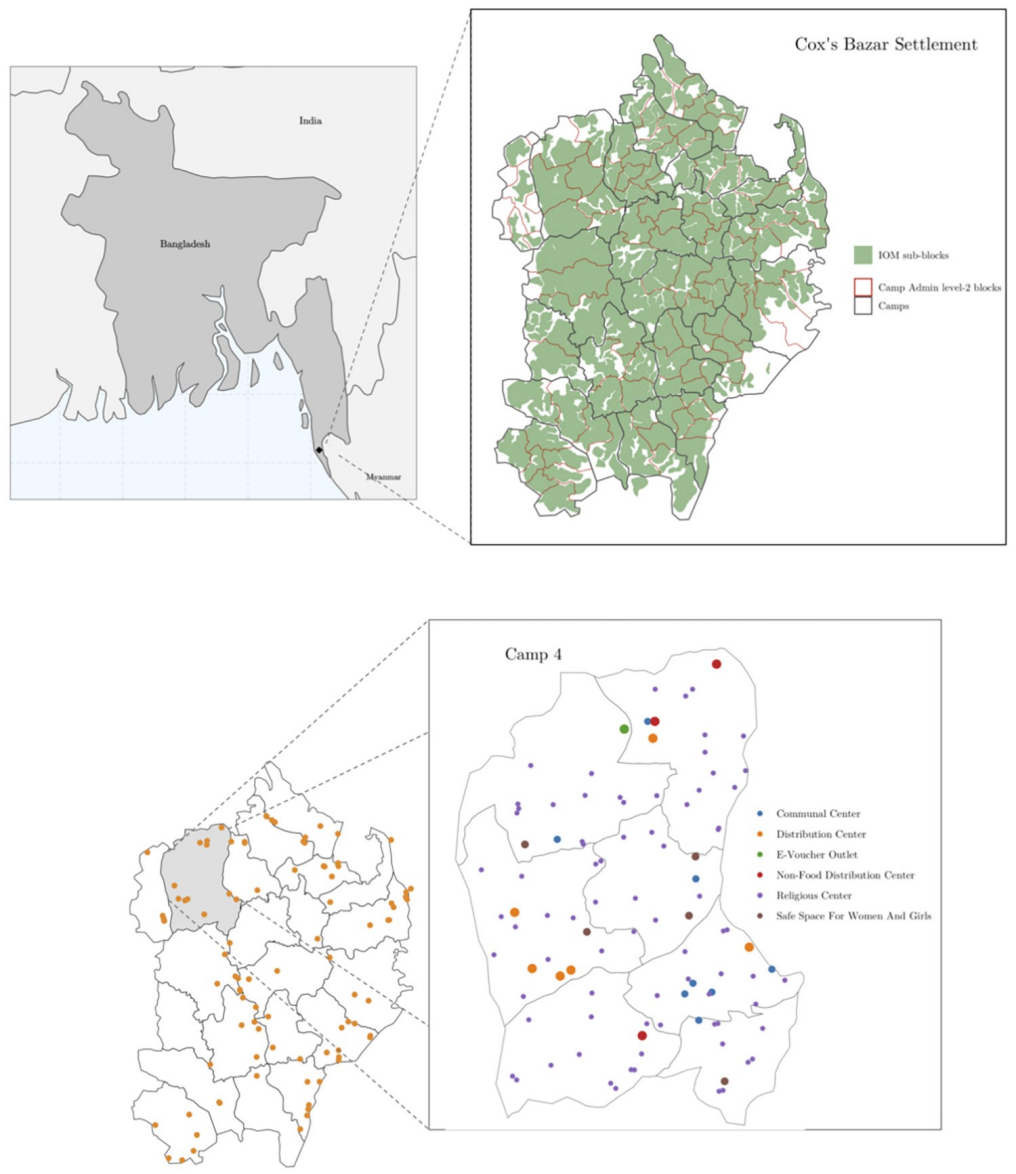
Digital twin geographic and location information. Upper left: Map of Bangladesh showing location of the Cox’s Bazar settlement. Upper right: Map of the modelled expansion site with three geographical layers. Lower left: modeled distribution centers. Lower right: Detailed view of Camp 4 showing six types of modeled locations. Basemaps from [22, 23].

The June model allows users to define three geographical entities in increasing order of granularity: regions, super areas, and areas [19]. For the most aggregate level (‘regions’) we select the camps which make up the Kutupalong-Batukhali Expansion Site. For the middle geographical layer (‘super areas’) we use the camp Admin level 2 blocks [20]; each camp contains 6–8 blocks. Finally, for the highest level of granularity (‘areas’), we use the sub-blocks as defined by the International Organization for Migration (IOM) [21]. Fig 1 shows the three different geographical layers used in our model of the refugee settlement in Cox’s Bazar.

#### 2.1.2 Demography

Once the geographical hierarchy has been built, we construct the virtual population. We initialize the population with age and sex attributes using statistical data from census records collected by UNHCR and the Government of Bangladesh for the camp Admin level 2 blocks (super areas) [24]. The number of residents in each IOM-defined sub-block (area) is assigned in proportion to the population of these areas [25]. We naturally capture the heterogeneity in population density and demographic attributes by ensuring that our digital twin reflects the distribution of residents at the sub-block (area) level, and the statistical age and sex characteristics of the camp Admin level 2 blocks (super areas).

Finally, national distributions of comorbidities by age and sex from Myanmar (the origin country of the PoCs) were used to assign comorbidities to the virtual population based on each agent’s age and sex. We assumed, for practical reasons, that an individual has at most one relevant comorbidity that would affect the probability of severe infection. Details on the estimated effects of comorbidities and the assignment process for comorbidities can be found in Section 2.2.2 and S1 Appendix.

#### 2.1.3 Shelters

Intra-family interactions create key transmission routes for infectious diseases. Correctly modeling family (we use the term ‘household’ interchangeably with ‘family’) and shelter compositions is therefore important to enable realistic reproductions of disease spread. We use data on the number of households at the sub-block (area) level as given by IOM [25] and data on the total number of residents in each Admin level 2 block (super-area) [24] to cluster individuals into households according to their age and sex, in order to create realistic demographic household structures.

Once we have constructed the households, we group households into shelters in which multiple households can live. In the Cox’s Bazar settlement, approximately 75% of the families share a shelter with another family [5] which results in an average shelter size of 7 persons. In general, shelters will have one or two rooms meaning two families may live in the same space, or may have a dividing wall between them, while mixing in communal areas.

#### 2.1.4 Learning centers

Learning centers refer to classroom settings for educating children and young adults [26]. Due to the large number of children in the settlement, and the limited number of educational facilities, children usually attend the centers for two hours a day, and several blocks of two-hour teaching sessions occur daily in each learning center [27]. We model these activities by assigning children and teachers to available learning centers in their camps, according to the centers’ proximity to the students’/teachers’ shelters. Only children enrolled in the education system are sent to learning centers in our model, and they attend one of four available two-hour time slots. The number of children who attend learning centers is chosen to match enrollment statistics collected at the camp (region) level, stratified by age and sex [26, 28–30].

#### 2.1.5 Dynamic locations

The shelter and classroom constructions described above capture interactions between static groups of people. We currently assume that household and shelter compositions are fixed, along with the learning center attended by each enrolled child. However, there are many other locations at which attendance and mixing are highly dynamic, such as aid collection stations or hand pumps and latrines.

Table 1 details the additional locations in the Cox’s Bazar settlement which we include in the model. We also model interactions between different shelters through family and individual visits. In Section 2.2 we discuss how we select which people visit these locations, and with what frequency, based on available research and literature.

**Table 1:** Classes of locations that simulated PoCs can visit in the model.

Activity	Type
Shelter Visits	Shelter
Distribution Center	Indoor
Non-Food Distribution Center	Indoor
E-Voucher Outlet	Indoor
Communal Center	Indoor
Safe Space for Women and Girls	Indoor
Religious Center	Indoor
Learning Center	Indoor
Hand Pump and Latrine	Outdoor
Play group	Outdoor

### 2.2 Simulator

The second stage of our modeling process involves designing the simulator which probabilistically models the social mixing and dynamic interactions of the virtual population. The digital twin forms the basis upon which the simulator is constructed. Each person in the model has the potential to move and interact with others based on individual and group dynamics which are derived from data. Since we model movement at the individual agent level, we have the ability to flexibly change all parameters used in the model and allow for different social mixing behaviors. In this section we provide an overview of the simulator set up. For additional details on the underlying methodologies and parameter choices please refer to S1 and S2 Appendices.

#### 2.2.1 Daily routine

**Fig 2:**
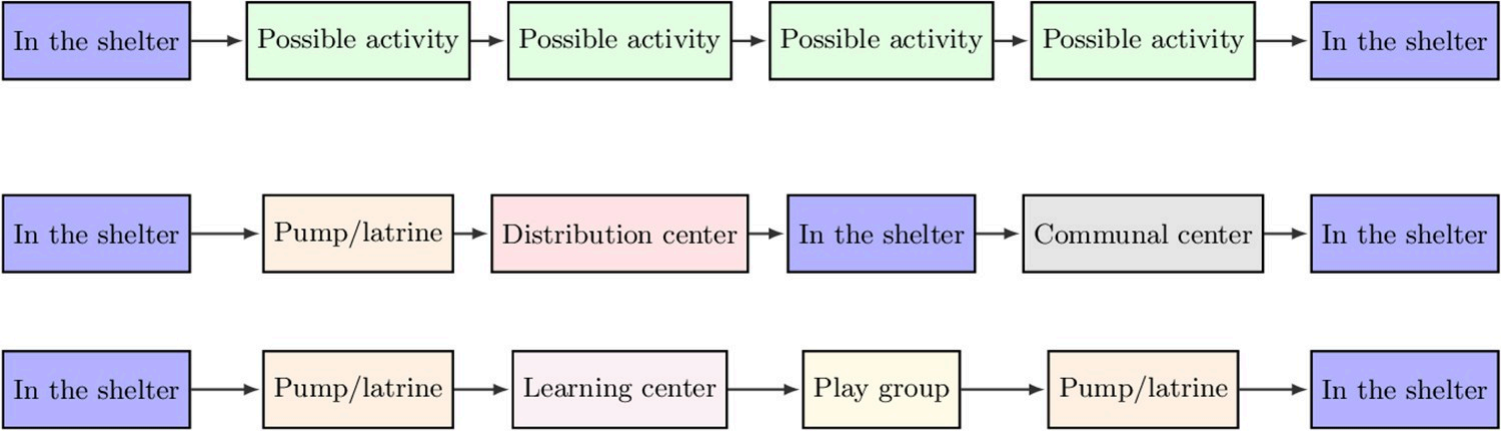
Top: Daily routine structure for individuals modeled in the simulation. We allow each individual to perform up to four possible activities per day (although this can be flexibly changed). If an activity is not chosen, the individual returns to their shelter. Middle: Example of a simulated day for an adult in the settlement. Bottom: Example of a simulated day for a child attending a learning center in the settlement.

To model the movement of individuals in the camp, we divide each simulated day into several time steps as shown in Fig 2. In each time step, an individual has a certain probability of doing one of several possible activities, during which they might interact with others who carry out the same activity in the same location contemporaneously. If no activity is chosen the agent will remain in their shelter. The statistical nature of this type of activity choice means that the routines for each individual change each day, with the exception of a few fixed activities such as sleeping in the shelter at night. This is a reasonable assumption given the operation of such settlements [31].

Simulated individuals begin the day in their shelters, where they can interact with others in their household, or other households which share the same shelter (see Section 2.1.3). After this time step, each day contains four opportunities for the individuals to undertake an activity in one of the locations listed in Table 1. The probability that an individual carries out a certain activity is determined based on their age and sex and available research on individual activity patterns (see S1 Appendix for more details on how these are derived from available literature). This implementation enables realistic modeling of how individuals behave as we can tailor the amount of time individuals spend in a given location based on demographic attributes of the population in accordance with available literature. This procedure also allows us to capture both local and inter-camp mixing.

#### 2.2.2 Disease

At each time step, different collections of individuals will inhabit the same space (e.g., a distribution center or play group) during which interactions and transmission can take place. The probability of disease transmission depends on various attributes such as duration of exposure, whether the individuals are indoor/outdoors and the type of contacts people might have. For further details see S1 Appendix and Aylett-Bullock *et al*. [19].

Once an individual has been infected, we assign them a health trajectory that controls the severity of their symptoms as a function of time. Each trajectory is characterized by the final outcome of the disease (whether the individual recovers or dies), the different stages through which the individual passes to arrive at that final outcome, and the duration of each stage. At present, we do not include the possibility of re-infection, after an individual recovers their susceptibility becomes zero.

**Fig 3:**
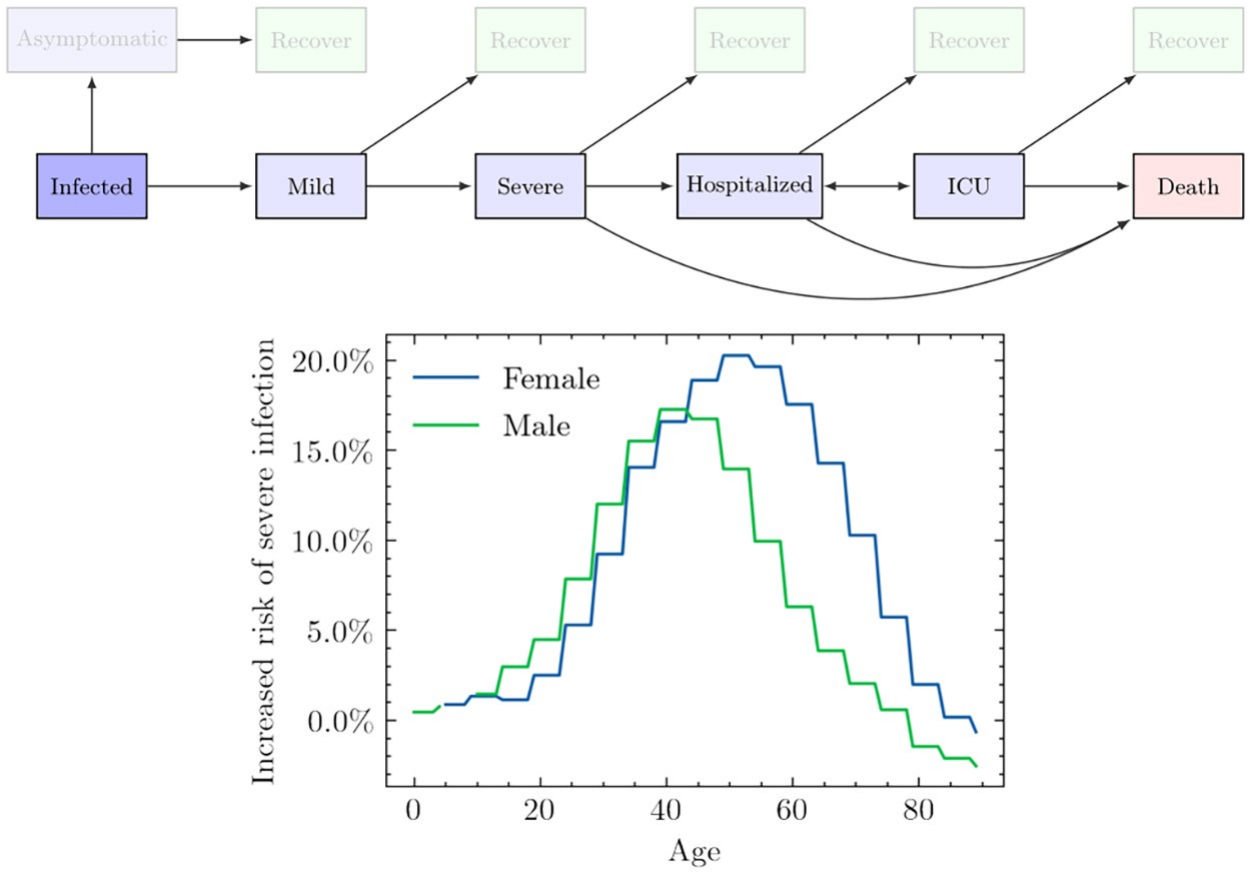
Upper: Modeled health trajectories. When an individual is infected, they might remain asymptomatic, or become symptomatic with symptoms likely to progress. In our implementation, the likelihood of each of these transitions is dependent on the age, sex, and/or comorbidities of the infected individual. As more data becomes available, additional factors can also be included. Lower: Severe infection rates adjusted for estimated comorbidities in the PoC population using UK data as a baseline. We show the increased risk of severe infection due to the presence of comorbidities, *r*_*c*_(age, sex), defined as, *P*^*Cox*^(severe | age, sex) = *P*^*UK*^(severe | age, sex)(1 + *r*_*c*_(age, sex)). Note that although *r*_*c*_(age, sex) decreases for the oldest age groups, *P*^*UK*^(severe | age, sex) increases exponentially with age and therefore the older an agent is, the more likely it is for them to develop a severe infection.

In Fig 3, we show the stages that we include, together with the possible trajectories defined by arrows. We incorporate the distinction between mild and severe infection to differentiate between people who show symptoms but are still well enough to leave their homes, and people who develop more severe illness that prevents them from doing so. An important factor that is difficult to account for, but which influences the probability of hospitalization, is the evolution and diversity of healthcare service seeking behaviour. Some survey data is available on how PoCs attend healthcare services in the event of severe illness [36], although we found respondents to be largely uniformly highly likely (over 95%) to seek care in this event. Despite this, increased data collection on how and when PoCs attend different healthcare services in the event of disease outbreak could be beneficial for future modeling efforts. However, even with available data on PoC behaviour, it is challenging to know exactly how to translate this into modeling work—for example, it is not well-understood for emerging diseases, such as COVID-19, to what extent the likelihood of death changes when an infected individual does not seek hospital care, even though the severity of their symptoms requires it. We therefore leave the inclusion of differing healthcare seeking behaviours for future work due to the lack of publicly available behavioural and clinical data for the specific case of COVID-19. Furthermore, the metrics used to measure intervention efficacy in this paper focus on infections rather than hospitalisation and death, meaning we are less sensitive to differing healthcare seeking behaviours.

#### 2.2.3 The effect of comorbidities on disease progression

An individual’s response to COVID-19 and other diseases can depend on the presence of illnesses such as diabetes, heart conditions, and conditions causing immune suppression [32, 37, 38]. To better reflect the specific evolution of the virus in the Cox’s Bazar settlement, we accounted for comorbidities which are assumed to affect the probability of severe COVID-19 infection.

Specifically, we allow the probability of following one of the disease trajectories outlined above to depend on age, sex, and comorbidity status. As a baseline, we use estimates from Aylett-Bullock *et al*. [19], derived from UK data, to determine the likelihood of each trajectory for any given case of COVID-19 conditional on age, sex, and the comorbidity distribution in the UK. We then adjust these likelihoods for the age, sex, and comorbidity status of each member in our simulated population (further details are found in S1 Appendix).

In Fig 3, we show the relative risk of severe infection after accounting for comorbidities in the settlement compared to the UK [32–35]. We define relative risk as the mean probability of a severe outcome (severe symptoms, hospitalization, or death) for a population with the comorbidity prevalence in the Cox’s Bazar settlement, divided by the mean probability of a severe outcome for a population with the comorbidity prevalence of the UK. On average, residents of Cox’s Bazar settlement are at higher risk of severe infection across almost all age groups except for the oldest cohorts, compared with the UK population; this difference is most prominent for ages between 20–70. The estimated increased risk of severe infection originates from a higher probability to develop comorbidities in the age ranges 20–70 for the inhabitants of the Cox’s Bazar settlement, compared to those in the UK. The oldest age groups in the settlement present a lower probability to develop comorbidities compared to the same age group in the UK, which we hypothesize to be the result of survival bias; the older individuals in Myanmar tend to also be those with a smaller number of comorbidities.

### 2.3 Data visualisation tool

Accurately communicating the detailed data and insights produced by complex models is challenging: e.g., a single figure can clearly show either a comprehensive overview or detailed plots, not both. Another communication challenge is that correct interpretation of data is often relative; changes in infection numbers only have meaning when we consider factors like the total population, the worst case scenario, and the best case scenario, so policymakers must consider this information in totality. Additionally, due to inherent uncertainty about the correct hyperparameter values, simulations may experiment with a grid of practicable values, generating a large amount of data that can be difficult to interpret and communicate.

People often turn to dashboards to present succinct views of large datasets. However, each of our simulations produces enough information to warrant its own dashboard, and allowing easy comparison across simulations is a significant visualization challenge. To this end, we built an interactive dashboard to accompany our simulations. While this dashboard has been tested and deployed with results from our simulations in the Cox’s Bazar settlement, our discussions in this paper will be more conceptual, with the aim of presenting this framework and the importance of developing tools for communicating results.

**Fig 4:**
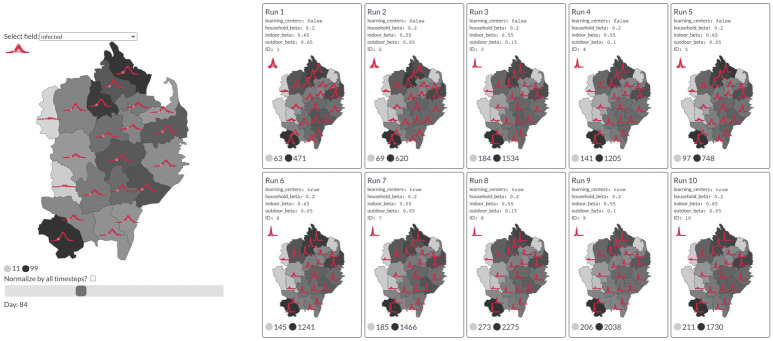
Left: An example from the dashboard showing daily infections by region for a single simulation. The red sparklines indicate the trend over the course of the whole simulation. The slider position marks 84 days after the start of simulation (i.e., the current time step). Darker regions indicate a higher infection count at this point in time. Right: Comparison of the daily infection rate of each region across 10 simulations of the “learning centers” scenario, colored by the peak infection rate seen in each region. Basemaps from [23].

Our dashboard is specifically designed to allow users to explore how different hyperparameter choices affect the outcomes of policy decisions, and helps translate key concepts between modelers and decision makers. For example, Fig 4 shows that the geographic distribution of infections does not seem to change by simulation, even though the overall height of the infection peaks varies significantly with the chosen parameters. More details on the tool and examples of comparisons between simulations are contained in S1 Appendix.

This dashboard is intended to serve as a collaborative tool in three main ways. For the researcher and data scientist, it enables rapid verification that the collective behavior of the agents aligns with expected real-world behavior, which can assist in identifying errors and debugging the model. For the policymaker, it provides an extensive view into the potential impact of different policy choices, enabling comparison across different assumptions. Finally, for those unfamiliar with the underlying base model it serves as a communication tool; the dashboard exposes the granularity of information that can be extracted from our agent-based model and instills confidence that the ‘best-case’ and ‘worst-case’ scenarios have been sufficiently analyzed.

## 3 Results

Once the digital twin and simulator have been set up, we are able to run simulations under different parameter configurations to model the possible effects of different operational interventions. Given the incompleteness of testing and case reporting data in the Cox’s Bazar settlement, we focus primarily on analyzing intervention efficacy through comparing the relative magnitudes of infection curves between various implementation conditions. Different models and approaches can account for different degrees and types of uncertainty making consensus on statistical predictions challenging even in more data rich environments. However, despite often highly variable predictions, consensus can often be reached on ranking intervention efficacy [39] which can be of interest for decision making.

The interventions presented in this paper are chosen based on those deemed most important by public health officials operating in the settlement according to an assessment of the short and medium-term needs including feasibility and timeliness. All interventions are compared with a baseline scenario which includes current policy decisions, such as the closure of certain venues and changes in the probability with which people perform certain tasks. S2 Appendix details the assumptions made for each activity that a digital person may participate in.

Interventions are implemented either through changes in the interaction intensity parameters in different locations (i.e. *β*^(*L*)^ parameters—see S1 Appendix), or through changes in the movement of digital individuals in the model. Limited COVID-related statistics means fitting the intensity parameters to data with a high degree of confidence is not possible. Therefore we estimate their values based on available literature (details on how we perform this estimation can be found in S3 Appendix). Indeed, the parameter values chosen when presenting the results of possible interventions are designed to explore a large region of parameter space and therefore aid in assessing the effects of model parameter uncertainties on scenario planning. Alongside this, stochasticity in the model can contribute towards the uncertainty of results, however, we find these uncertainties to be negligible for our model (see S3 Appendix).

For simplicity, in the baseline model we assume that all symptomatic individuals with mild symptoms self-quarantine in their shelter with a low compliance of 30% (each individual has a 70% chance of breaking quarantine at each time-step) to account for difficulty in communicating quarantine procedures, as well as the inability of many individuals to properly quarantine given certain basic needs [31]. There are limited contract tracing efforts currently ongoing in the settlement, however, these are not included in the model given their more recent introduction. As mentioned in Section 2.2.1, those with severe symptoms are by definition required to stay in their shelter in line with [19]. Given the makeup of the shelters, quarantining individuals will not leave their shelter but may still interact with those in their shelter. All models are seeded with 88 infected individuals across a variety of geographic regions based on data collected on the 24th May 2020 [40]. The baseline model assumes a moderate transmission scenario (*R*_0_ ≈ 2.0 − 3.0) as estimated in Truelove *et al*. [17]. Details of the rationale and precise procedure for the initial seeding is given in S3 Appendix.

The primary metrics we use to assess and compare scenarios include the time to infection peak, the height of the peak, and the total number of infected. Time to infection peak and the height of the peak are important because they serve as a proxy for how quickly a settlement’s response capacities will be overwhelmed and to what degree; all else equal, responders would prefer a slower rise in infections in order to have more time to prepare for a surge. Total infections are important because they are a proxy of the settlement-wide impact of COVID-19.

### 3.1 Isolation centers

In many countries, those with symptoms which are not yet severe enough to require hospitalization are encouraged to stay at home and self-quarantine. In the case of settlements such as that in Cox’s Bazar, the density and living conditions of the residents mean that avoiding contact with family in the home environment is not possible, and individuals frequently have to leave their shelter to use facilities such as hand pumps and latrines. In an attempt to better enable the isolation of symptomatic individuals, public health officials in the settlement set up isolation and treatment facilities to house those who have tested positive for COVID-19 but do not require hospitalization [41, 42].

**Fig 5:**
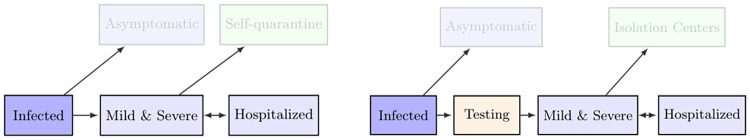
Isolation center scenarios. Left: Home-based care scenario where mild and severely symptomatic PoCs self-quarantine in their shelters up to a compliance factor. Right: Scenario where mild or severely symptomatic individuals go to isolation centers up to a compliance factor.

We modelled two scenarios: (a) in which patients with mild and severe symptoms (not requiring hospitalization) self-quarantine and are treated at home (referred to as “home-based care”); and (b) in which symptomatic patients go to isolation and treatment centers regardless of symptom severity up until they need to be hospitalized (we refer to this scenario as “treatment center scenario”). A schematic of these scenarios is given in Fig 5.

To explore different was in which treatment centers are used in scenario (b), we varied the average time delay between symptom onset and isolation—this is designed to encapsulate the delay between a symptomatic individual developing symptoms and presenting themselves for testing, the time taken to process the testing, and the the time spent in the isolation center. The first of these is a clear behavioural assumption—to assess the effects of particular scenarios in reality, further studies could be completed to validate the true value of the time delay. Further details on these parameters are given in S1 Table.

**Fig 6:**
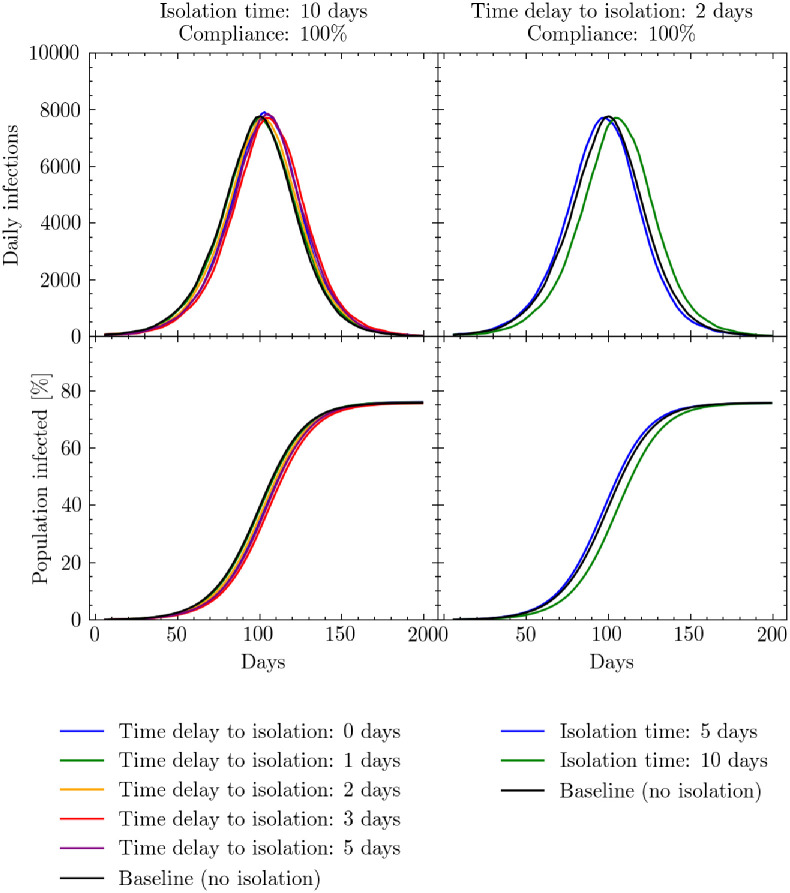
Isolation center simulation results. Simulated daily (7-day rolling average) and cumulative infections measured in days since the beginning of the simulation. Left: the effects of varying the mean time delay to attend an isolation and treatment center from symptom onset relative to the baseline home-care scenario while time spent in the isolation and treatment center is fixed at 10 days. Right: the effects of varying the time spent in an isolation and treatment center while keeping the mean time delay to the center fixed at 2 days. In both scenarios the compliance rate that people present themselves for isolation is set too 100%. See S1 Table for a presentation of the cumulative number of infections, peak intensity and peak timings for these scenarios.

Fig 6 presents the results of our simulations. We first simulate, the effects of varying the average time delay between an individual developing symptoms and presenting themselves at an isolation and treatment center with a. To disentangle parameter dependencies, we fixed the time spent in isolation to 10 days and the compliance level for an individual to go to the centers at 100%. This presents a best case scenario for the isolation of individuals. Here, we see that varying the average time delay to isolation had little effect relative to the baseline home-based care scenario (and is largely within the stochastic limit of the model—see S3 Appendix).

Secondly we simulate the effects for varying the time spent in the isolation center, while fixing the compliance to 100%. Here we also fix the average time delay to isolation to 2 days to represent an optimistic yet realistic scenario. Again, we see this has little effect on the daily infection rate relative to the home-based care scenario.

The reason for this similarity between the treatment and home-case scenarios is likely due to the infectiousness profile presented in in S1 Table. Given the likely rapid transmission of infection between those residing in the same shelter (see S3 Appendix for a breakdown of location of infections in the baseline model), the majority of infections have likely taken place before symptom onset. To test our sensitivity to this profile, we examined the effects of shifting the peak of the infectiousness profile ± 10% but this yielded little difference in results.

The results presented in this section suggest that encouraging home-based care for individuals with mild symptoms may not have a major negative impact on the number of daily infections as was the initial concern and therefore isolation center beds could be preserved for only those requiring hospitalization.

### 3.2 Mask wearing

Widespread adoption of face masks has the potential to significantly reduce the transmission of COVID-19 [43, 44]. In the settlement, surgical and cloth masks have been distributed to many PoCs, with the majority being of the latter type [45, 46]. However, with limited supply chains, surgical masks are often being washed and reused which can significantly alter their efficacy [31, 47, 48]. The overall success of mask policies are contingent on both the efficacy of the masks themselves and compliance with mask wearing. To test the potential effect of mask wearing we simulate the use of masks with different efficacies in all settings outside the shelter, with the exception of play groups, and with variable rates of compliance. More details on mask wearing efficacies and our parameter choices can be found in S1 Table. As in [19], this is encoded through a change in the interaction intensity parameters which are adjusted according to:

$$\begin{array}{c} \beta^{*(L,g)}=[1\;-\;C^{\left(L\right)}\cdot E]\beta^{\left(L,g\right)}, \end{array}$$ (1)

where β^*(*L*,*g*)^ is the new interaction intensity parameter, *C*^(*L*)^ is the compliance with correct mask wearing in a given location *L*, and *E* denotes the mask efficacy. Efficacy is defined as a function of the mask material, as well as any degradation through incorrect reuse and washing. While we could also specify certain geographic regions in which mask wearing takes place, at present we assume that the location specific compliance factor, *C*^(*L*)^, refers to all relevant locations in the settlement.

**Fig 7:**
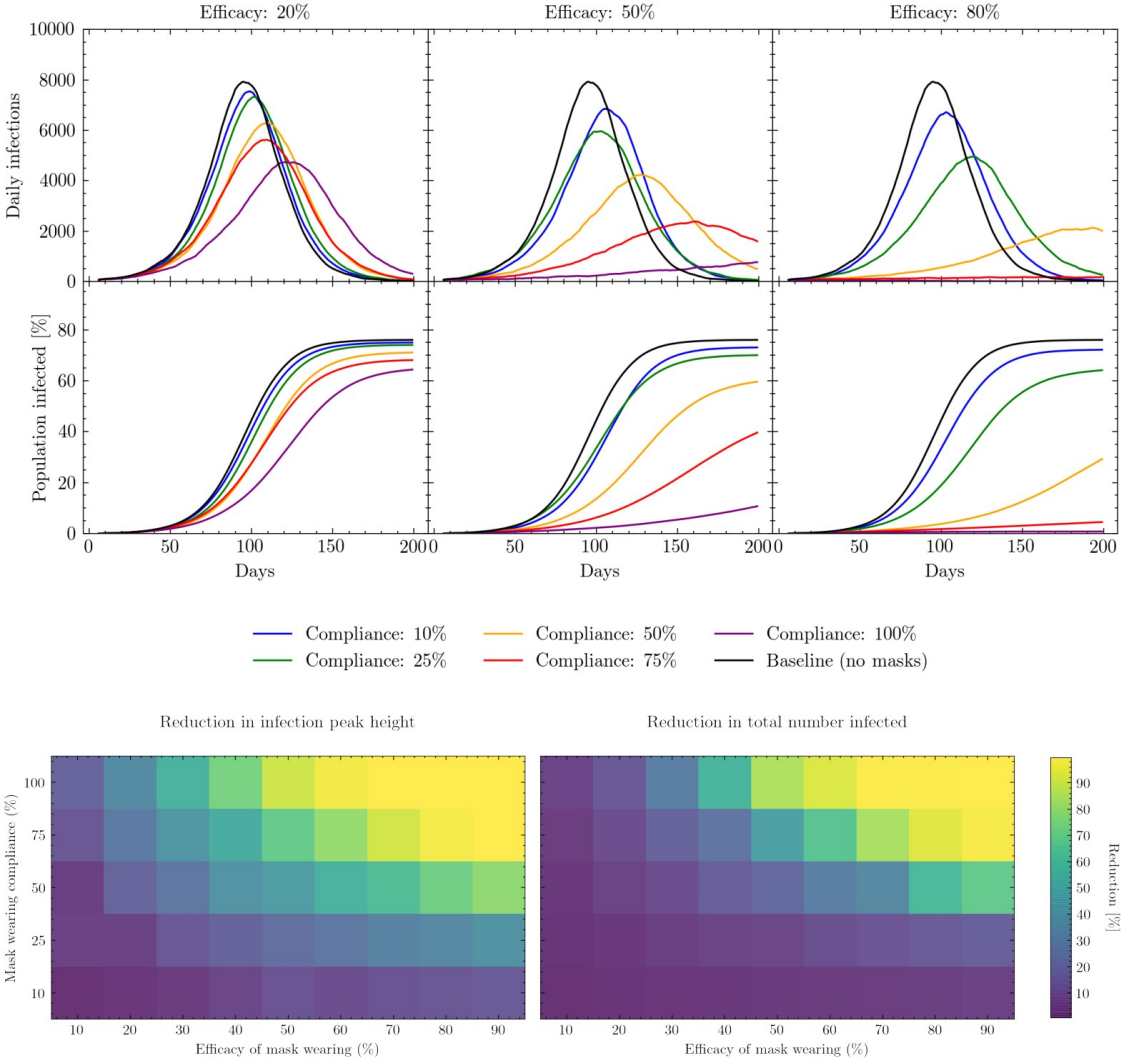
Mask wearing simulation results. Upper: Simulated daily (7-day rolling average) and cumulative infections measured in days since the beginning of the simulation. Results show the effects of varying the compliance with mask wearing in different locations under different assumptions regarding mask efficacy. The baseline model is the scenario in which no masks are worn. See S1 Table for a presentation of the cumulative number of infections, peak intensity and peak timings for these scenarios. Lower left: percentage change in daily infection peak height as a function of mask wearing efficacy and compliance relative to the peak height of the baseline model. Lower right: percentage change in total number of infections up to a fixed point in simulated time as a function of mask wearing efficacy and compliance relative to the total number of infections simulated by the baseline model. The baseline model assumes no masks are worn.

Fig 7 (upper) shows the simulated effect of mask wearing on the daily number of infections as a function of compliance and mask efficacy. When mask efficacy is low, e.g. of the order of 20%, relative changes in compliance have a comparably small effect on the total proportion of the population infected. As the average efficacy of the masks increases, these changes in compliance can have a clearer effect, yet we see that further increases in average mask efficacy beyond the 50% level may have diminishing returns in realistic scenarios. For example, obtaining masks with average efficacies greater than 50% (which we assume to be equivalent to correctly wearing a single-layer cotton mask [43, 49, 50]) may be challenging and costly, especially when efficacy is also a function of correct mask useage, and the resources required to achieve this may be greater than the gain in transmission reduction. Overall it is important to note that even though the majority of infections take place in the shelter (see S3 Appendix), partially effective masks have the chance to prevent many of those infections which are key to bringing the virus back into the home.

Fig 7 (lower) illustrates how varying both compliance and efficacy affects both daily infection peak height and the total proportion of the population infected. As expected, both the height of the daily infection peak and the total number of people infected decline as mask efficacy and compliance increase. However, this figure also shows that peak height and total number of infections respond differently to changes in compliance or efficacy: peak height reduces faster than the total number infected as compliance and efficacy increase (i.e, it is more sensitive to changes in compliance and efficacy).

In the case of mask wearing specifically, it is informative to know where to utilize already scarce resources—on increasing compliance or on increasing the average efficacy of the mask. From Fig 7 (lower) we also see the relative sensitivity to the compliance and efficacy parameter individually. For example, assuming an attainable efficacy of 50% [49] with a compliance rate of 50%, it can be seen that an increase in compliance by 25% (which could be encouraged through e.g. risk communication and community engagement (RCCE) exercises) has a similar effect to increasing mask efficacy by 30% on the daily infection peak height. Such considerations are operationally important as the wider use of lower efficacy masks which can be homemade and reused, thereby increasing compliance, may be considerably easier than importing large quantities of higher quality single use masks, such as surgical masks. Ongoing monitoring of mask wearing compliance could be utilized to dynamically adapt resource allocation to ensure they are used most efficiently.

### 3.3 Opening learning centers

Learning centers in the settlement have been closed since March 2020 in an attempt to mitigate the spread of COVID-19 [51]. Although opening learning centers imposes a risk of infection within the classroom, the closure of learning centers has serious consequences on the educational development of the children and may also have negative consequences on the epidemiological development of the virus: when children are not in school, they participate in various activities such as assisting with aid collection, going to communal centers, or meeting up with other children and playing in groups outside which all serve as additional channels for intermixing [26, 29, 30]. Indeed, since the learning centers have been closed, settlement officials have observed an increase in children meeting up and playing in small groups [31].

To simulate the possible effects of opening the learning centers we allow all children enrolled in the education system to go to school each day as described in Section 2.1.4. To avoid multiple concurrent parameter variations, in previous intervention scenarios we fixed the interaction intensity parameters as the relative sizes of these parameters were not as important as others to understanding the potential effects of the intervention. However, in the case of learning center opening the relative intensity of interactions in the indoor and outdoor environments becomes key since when children are not in learning centers they are predominantly in outdoor environments.

Currently, it is unclear how intense interactions in learning environments might be relative to interactions with other children outside. To account for this unknown relationship, we varied the ratio of the interaction intensity in both indoor and outdoor settings to the interaction intensity in shelter settings while preserving the shelter-indoor-outdoor intensity hierarchy described earlier in this section. See S1 Table for more details on the parameters choices for these scenarios.

**Fig 8:**
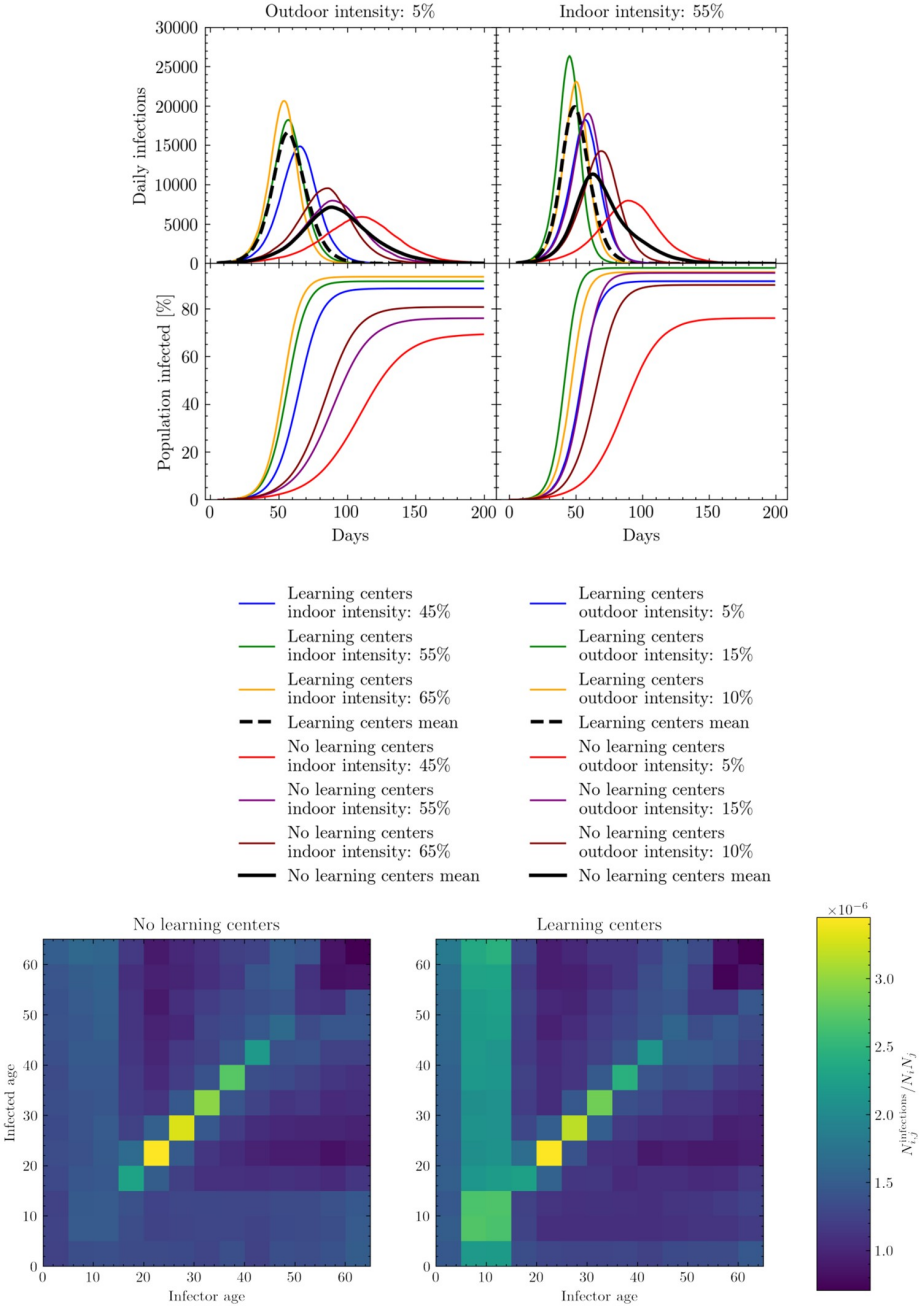
Learning center opening simulation results. Upper: Simulated daily (7-day rolling average) and cumulative infections measured in days since the beginning of the simulation. Results show the effects of varying indoor (left) and outdoor (right) interaction intensity parameters relative to the interaction intensity parameter set for the shelter. These intensity parameters are varied in both the baseline models and those with learning centers open. See S1 Table for a presentation of the cumulative number of infections, peak intensity and peak timings for these scenarios. Lower: Simulated number of cumulative infections in one age group produced by another age group normalised by group sizes. We assume the baseline interaction intensities here.

Fig 8 (upper) shows the effect of opening learning centers. The left set of panels demonstrate that varying the indoor intensities can have a non-trivial effect on the progression of the virus through the population, although the two scenarios—opening the learning centers or keeping them closed—remain well distinguished from each other in both peak height and timings. The right set of panels show that varying the outdoor intensity can have significant effects on both peak height and location, with some scenarios less well distinguished. This difference occurs as, with the exception of learning centers, indoor locations outside of the shelter environment are much more irregularly visited by children in comparison to the rate at which they meet up outdoors with each other (see S2 Appendix for more details). Despite this, the mean values of the scenarios clearly demonstrate different epidemiological trends.

Although opening learning centers may increase both the cumulative number of infections and rate of disease spread, it might be expected that this growth observed in Fig 8 (upper) is predominantly constrained to the younger age groups. However, in Fig 8 (lower) we see that although opening learning centers does increase the chance of children being infected significantly, this increase in infections rapidly breaks out of age-strata likely due to mixing in often inter-generational shelter settings. This, coupled with the increase in the cumulative number of infections, suggests that by opening learning centers the virus will also likely infect individuals who were previously naturally shielded by a form of herd immunity.

### 3.4 Mitigation strategies for opening learning centers

Reopening learning centers is a priority because the longer the learning centers remain closed, the longer children in the settlement go without school and risk having their educational development stunted. In Section 3.3 we found that the opening of learning centers may facilitate the spread of COVID-19. However, the simulations described were only designed to account for the effects of opening learning centers under the same conditions they operated in before the virus was circulating. In this section, we explore several possible strategies for opening learning centers with additional transmission mitigation strategies.

We model three possible strategies: i) adjusting the regularity with which children in the settlement attend learning centers, and therefore limiting their mixing in these environments; ii) opening more learning centers in alternative spaces; and iii) introducing specific measures to lessen the interaction intensities in the learning centers. This final strategy could consist of combinations of physical distancing in classrooms, mask wearing, increasing classroom ventilation, and more thorough cleaning and hygiene. For clarity, to limit the number of concurrent parameters being varied, we compare all these scenarios to our baseline with no learning centers open and fixed intensity ratios. See S1 Table for more details on the parameters choices for these scenarios.

**Fig 9:**
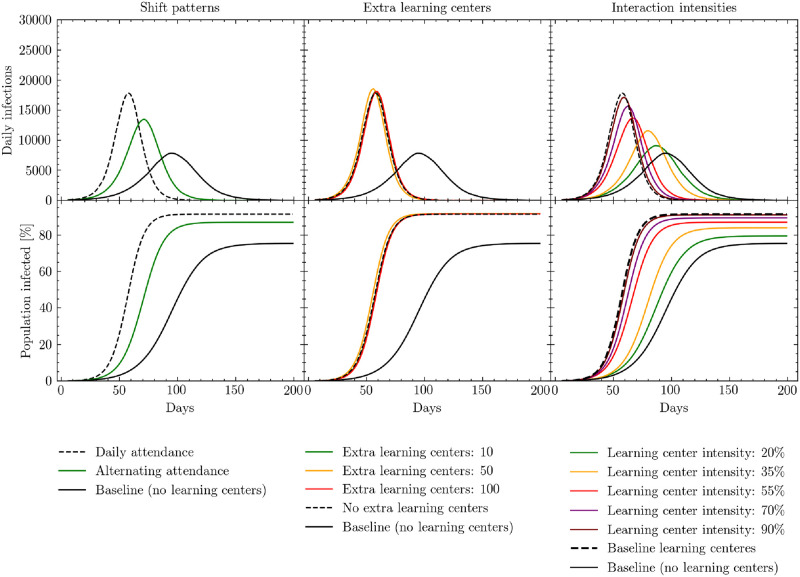
Simulated daily (7-day rolling average) and cumulative infections measured in days since the beginning of the simulation. Black solid lines represent the baseline policy in which learning centers are closed. Black dashed lines represent the policy in which learning centers are open with no additional mitigation strategies. Results show the effects of opening learning centers under three conditions: adjusting the regularity with which children attend learning centers (left); opening additional learning centers (middle); reducing interaction intensities in the learning centers through strategies such as physical distancing, masks, and improved ventilation (right). Note that in the middle panel, the green line overlaps almost precisely with the baseline. See S1 Table for a presentation of the cumulative number of infections, peak intensity and peak timings for these scenarios.

The first mitigation strategy we test is changing the regularity with which children attend learning centers. Normally, children enrolled in the educational system are expected to attend their learning center each day; however, by halving the attendance rate and having children only attend once every other day, mixing between different children can be reduced. This intervention would also better enable physical distancing as the number of students in each class is halved. The results of simulating this intervention are presented in the left panels of Fig 9. We see a significant delay in the number of days to peak infection, and a lower percent of the population infected, as a result of alternating the days on which children attend learning centers. While this strategy is clearly effective, we may see additional benefits by combining it with other mitigation strategies.

Second, we investigate the possible effects of increasing the number of learning centers in the settlement. To implement this strategy, we first distribute all children to the existing learning centers and then rank the learning centers by those with the biggest class sizes. These large classes pose a higher risk of viral transmission between households, and therefore present a particular danger. Once we have identified the centers with the largest classes, we add another learning center in the same location to our model, thereby mimicking the strategic opening of new learning centers to effectively halve the class sizes of the most crowded centers. Once these new learning centers have been added to the model, we redistribute children from the crowded classrooms to the new centers. As shown in the middle panels of Fig 9, the effect of opening a limited number of additional learning centers is negligible. Relative to the approximately 1,200 learning centers already operating in the settlement, opening 10–100 new centers (which we chose to be the upper-end of a feasible implementation but may already be logistically challenging) does not alter the mixing of children enough across the settlement to have a significant impact.

Finally, we examine the effects of changing the intensity of interactions within the learning centers, while keeping the other indoor, outdoor, and shelter interaction intensity parameters fixed. The range within which we vary these interaction intensities (20–90%) corresponds to the changes in interaction intensities that result from various combinations of physical distancing, mask wearing, and increasing ventilation.

Fig 9 shows the simulated effects of reducing interaction intensity in the learning centers relative to the baseline learning center intensity described above. Reducing learning center interaction intensities has the potential to significantly affect the height and positioning of the daily infection peak, as well as the total number of individuals infected. When learning center interaction intensities fall below 20–35%, daily infection statistics begin to approximate the scenario in which learning centers remain closed, thereby almost completely mitigating the effects of opening the centers. As discussed in Section 3.2 and S1 Table, the upper end of this relative intensity range could correspond to enforcing mask wearing alone if compliance and the efficacy of the masks worn are high or a combination of mask wearing and physical distancing. The lower end may correspond to the combination of physical distancing in classes, mask wearing and improved ventilation [43, 44, 50, 52, 53]. With respect to this latter combination, it is important to note that ventilation options in schools vary with the type of classroom, e.g. some learning centers are built from bamboo allowing for more natural air to flow, while others appear to be smaller, concrete rooms [54, 55]. In enclosed settings, ventilation could consist of opening windows and doors as well as using electric fans to increase air flow.

In summary, our simulations suggest that implementing a combination of mask wearing, physical distancing, and improved ventilation can significantly decrease the number of infections and potentially make it possible to open learning centers safely. While physical distancing may not be possible in classrooms given the current space available [56], this could be enabled by the reduction of class sizes induced through alternating attendance.

## 4 Discussion

Modelling the effectiveness of different operational interventions is important for future planning purposes. In a refugee settlement, implementing such interventions requires significant advance mobilization as well as operational and financial support. By simulating the possible effects of operational interventions prior to their introduction, and incorporating the results of these simulations into decision making processes, intervention priorities can be better identified.

In this paper we present an agent-based modeling approach, adapted from the June framework [19], to simulate disease spread in refugee and IDP settlements. The movement of people and their interactions are modeled at the individual level, with parameters informed by open-source datasets, empirical observations and recent research literature. Our approach first consists of building a ‘digital twin’ of the settlement in which the geographic layout is defined. Virtual individuals are included into the model with different demographic attributes mimicking real world statistics and family and shelter structures are reproduced. Locations in which individuals may interact are also included, such as learning spaces, distribution centers or hand pumps and latrines. Secondly we design a simulation engine which captures what people in the model do during the day, how they interact and how diseases may be transmitted. This underlying structure can then be used to model different operational interventions by altering the movement and interactions of different subsets of individuals in the model, or by closing certain venues. Finally, we present a dashboard designed to present the multiple insights and uncertainties inherent to this modeling approach which can serve as a shared tool for conversation and iteration between modelers and decision makers.

This work focuses on the spread of COVID-19 in the Cox’s Bazar refugee settlement in Bangladesh, although the approach is designed to be generalizable to other settings. Given incomplete testing and case statistics, we have focused on modelling the relative effects of various operational interventions on key statistics such as the daily infection rate, as opposed to producing precise forecasts. The interventions presented in this paper are chosen based on those deemed most important by public health officials operating in the settlement according to an assessment of the short and medium-term needs including feasibility and timeliness. However, there are also additional, more nuanced, questions about these interventions which could be investigated using our approaches, and we leave this to future work.

We analyzed the possible effects of alternate home-case delivery mechanisms, mask wearing based on compliance and the type of masks worn, and (re)opening learning centers under various scenarios. Our findings suggests that the isolation of people with mild to severe symptoms will likely have little effect given the assumed infectiousness profile of symptomatic individuals. Mask wearing, however, is found to be have potentially large positive effects, mitigating significant proportions of disease spread if worn in all indoor locations with the exception of shelters. For example, cumulative infections over the period of the simulation are reduced by 50% when half of the population comply with wearing cotton-type masks, and are reduced further when increasing compliance or mask efficacy. Alternatively, the opening of learning centers could present challenges, with the risk of increasing the growth rate of the epidemic. However, we also explore several strategies to opening learning centers, which if used in combination (such as increased ventilation, mask wearing and reducing class sizes), could greatly mitigate many of these risks.

One of the main limitations of this work so far has been the possible validation of model predictions with real world data—since case and testing data availability has also been limited. Our approach to understand the potential impact of interventions has been simulating the effects of interventions as if they were in place from the beginning of the simulated period. If required, however, in the event that more precise data becomes available, we expect to be able to perform further retrospective validations of the results by leveraging the flexibility of the model which can be fitted to historical trends, enabling the provision of future forecasts, as well as the simulation of different sequences of measures being implemented at different points in time (see example of this in [19]). Indeed, a serological study has been carried out throughout December 2020 in the Cox’s Bazar settlement which could serve as a key source of data for fitting, evaluating and constraining our modeling approach. In future work, we also plan to assess the impact of various vaccine distribution strategies in these settings.

Alongside up-to-date testing data for model validation and fitting, additional data on healthcare seeking behaviour in response to epidemic outbreaks would also be highly beneficial. This would allow modeling works to better factor in cultural differences in seeking different kinds of care, as well as the impact of potential misinformation and disinformation spreading throughout populations which could affect compliance with interventions. To fully utilize this, better clinical data on the effects of different healthcare seeking behaviour is critical. Further, we make the assumption that comorbidity prevalence in the settlement population is comparable to the country of origin (here assumed to be Myanmar), yet this is a simplification. Better data on comorbidities of the specific population in question, alongside a deeper understanding of the clinical ramifications, could significantly improve the accuracy of hospitalization and morality statistics. Finally, ongoing data collection on compliance levels with interventions would allow for the honing of modeling works to more specific scenarios and reduce the number of free parameters.

In any modeling approach it is important to tailor results and outputs to the specific environments and questions which need to be answered by decision makers. The approach presented in this paper has been developed in close collaboration between modelling teams and those operating in the Cox’s Bazar settlement. Research questions and operational scenarios have been defined jointly by the different teams involved in the project. In fact, we have found that the development of the data visualisation tool plays an important role in helping translate between groups with a wide range of expertise. It is crucial that public health specialist and decision makers have full understanding and ownership of the results of any modeling work. With this work we hope to encourage future multidisciplinary modeling efforts to engage fully with end users to ensure meaningful discussions take place and decisions are taken informed by the best possible science.

While the focus of this work has been on intervention planning during an evolving outbreak, these methods and techniques are applicable to future epidemics and different diseases. Modeling work can often be reactionary, however, through the use of scenario planning strategies such as those introduced in this paper, we hope to inspire further efforts with a focus on anticipatory action. Indeed, the results from such work could not just help for contingency planning, but also incorporated into designing settlement layouts to attempt to mitigate disease spread before it reaches epidemic levels.

## Ethics

This research has been designed and conducted following relevant data privacy and data protection principles and processes, including UNHCR data protection policies and guidelines, as well as the UN principles on Personal Data Protection and Privacy, and the UNSDG Guidance Note on Big Data for the 2030 Agenda: Ethics, Privacy and Data Protection. Data used for building the digital twin come from statistical data, other open datasets and anonymous and aggregated survey data used collected by UN agencies as cited throughout this document.

## Supporting information

S1 Appendix Methods. Detailed description of methods and algorithms used in the construction of the model.
(PDF)

S2 Appendix Digital twin and simulation parameters. Parameters, and their accompanying sources, used in the constructing the digital twin and simulation.
(PDF)

S3 Appendix Seeding, intensity parameters, and the baseline model. Description of the initial seeding and intensity parameter settings for simulation runs as well as details on the baseline model, and stochasticity.
(PDF)

S1 Table Intervention details. Additional details and discussions on exact parameter choices for certain operational interventions.
(PDF)
