# Supplementary material for: Operational response simulation tool for epidemics within refugee and IDP settlements: A scenario-based case study of the Cox’s Bazar settlement
Source: PLoS Comput Biol. 2021 Oct 28;17(10):e1009360. doi: 10.1371/journal.pcbi.1009360 (PMC8553081; doi:10.1371/journal.pcbi.1009360)
Supplement: S1 Appendix — Detailed description of methods and algorithms used in the construction of the model. (PDF) [file pcbi.1009360.s001.pdf]

S1 Appendix

Methods

This appendix provides additional details regarding the construction of the agent-based model used to simulate the spread of COVID-19 in the Cox's Bazar refugee settlement in Bangladesh.

Digital Twin

Geography

To define the three geographical levels we use shapefiles provided by UNHCR for both the camps and the admin level 2 blocks [1], as well as shapefiles from IOM for the sub-blocks [2], we encode the geographical hierarchy by assigning camp Admin level 2 blocks to the camps with which they overlap the most, and by assigning the IOM sub-blocks to the the Admin level 2 blocks with which they overlap the most.

Shelters

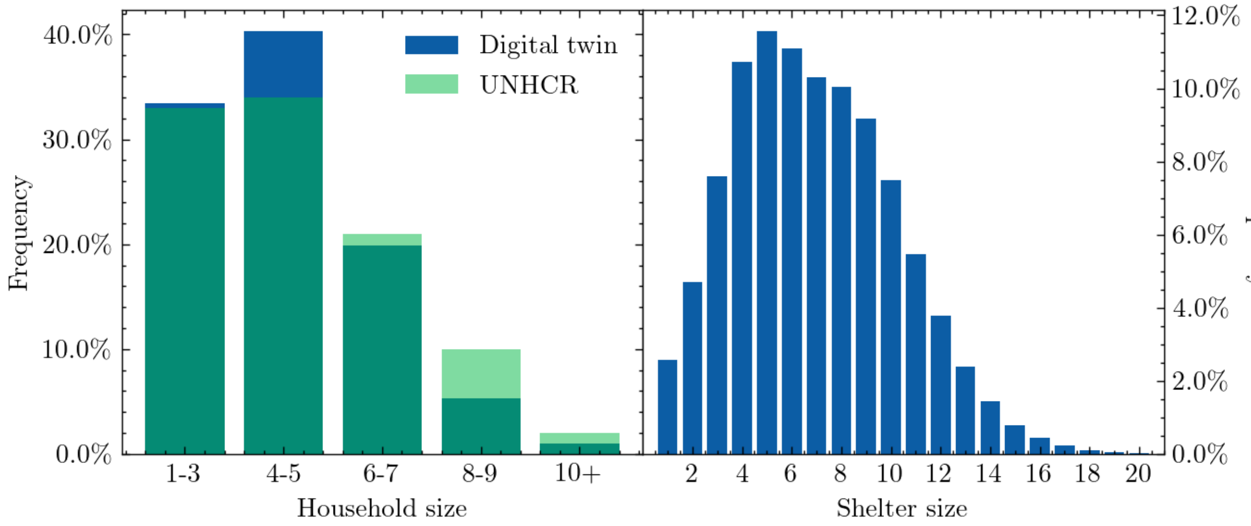

Fig 1. Left: Household size distribution comparing the UNHCR data with the digital twin of the Cox's Bazar settlement, where the binning has been chosen to match UNHCR's reporting structure. Right: Shelter size distribution from the digital twin.

We use data on the number of households at the sub-block (area) level as given by IOM [3], and data on the total number of residents in each Admin level 2 block (super-area) [4], to cluster individuals into households according to their age and sex to create realistic demographic household structures.

To account for data mismatches between the two datasets, we rescale the IOM's household statistics so that the total number of residents estimated based on these

household counts matches the total number of residents recorded in UNHCR's census, at the Admin level 2 block (super-area) level. Once we know the (rescaled) target number of households for a given sub-block (area), we construct them by sampling each household's size from the global household size distribution reported by UNHCR [5]. Each digital household is then populated according to the following algorithm:

1. We first allocate one adult (a person older than 16 years old) to each household, if available.
2. We iterate over all the non-full households, allocating one child (a person younger than 16 years old) each time until all children belong to a household.
3. For each non-full household, we choose an adult from the unallocated adult population by picking a person of the closest age to the current household adult resident, and of the opposite sex, if available.
4. The remaining adults are randomly distributed into households that still have space.

This algorithm naturally captures both single-headed households and child-headed households, as well as multi-generational families all of which are important for both protection concerns and disease spread.

In the left panel of Fig 1 we show a comparison between UNHCR data and the household distribution for the Cox's Bazar settlement derived from our model. The distributions do not match perfectly due to the introduction of uncertainty when rescaling the number of households. Nonetheless, the two distributions are in reasonable agreement, with UNHCR reporting an average household size of 4.6 and our model producing a mean size of 4.4 (see the left panel of Fig 1).

After clustering households into shelters the resulting shelter size distribution is plotted in the right panel of Fig 1. We note that, even though we underestimate the number of larger households (8-9 people), we do not necessarily underestimate the right tail of the shelter size distribution as we assume that all households are equally likely to share a shelter, while in reality bigger households are probably more likely to couple with smaller households. Furthermore, the particular realisation of the household population is randomised for each simulation, so the tail of the particular sample distribution changes for each run. We do not observe any noticeable effects of these fluctuations on the overall infection rates (see **??**).

Learning centers

We place the learning center into our model according to latitude/longitude coordinates derived from the Inter Sector Coordination Group (ISCG) [11]. Children are assigned to a daily time slot in a specific learning center, during which - from a modelling perspective - they have the potential to interact with other children in the class, as well as their teacher. We assume that each learning center offers four two-hour teaching blocks per day. In our model, each classroom is assigned one teacher who is drawn randomly from the population in the area in which the learning center is located, and only children enrolled in the education system in the camp are sent to school (see Fig 2). Each enrolled child will attend the learning center closest to his/her shelter, unless that learning center has reached a maximum capacity of 35 pupils per shift, in which case the child will go to the next closest learning center. If the nearest 50 learning centers are all full, one of them is picked at random.

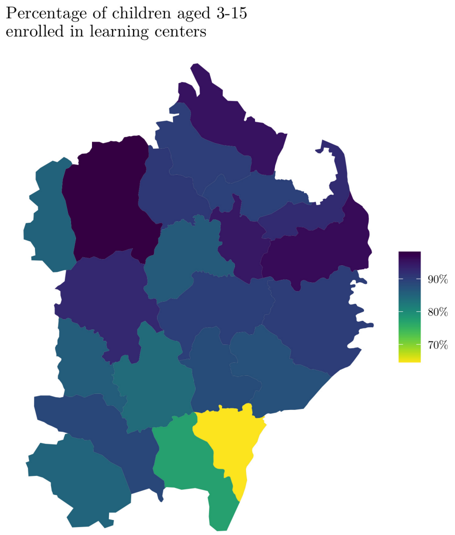

Fig 2. Enrollment of children aged 3-15 years old in each camp as a percentage of the total number of children living there [6–9]. Basemap from [10].

Dynamic locations

Using data collected by the ISCG [11], we place the dynamic locations using latitude/longitude coordinates as shown in Fig **??**. Hand pumps and latrines are distributed to sub-blocks (areas) based on statistics detailing the number of individuals sharing such facilities [12], while play groups are dynamically created by randomly drawing together groups of children from neighboring shelters.

Each shelter is linked to up to 3 other shelters, and each member of a shelter can visit one of its linked shelters during a simulation time step.

Simulator

Daily routine

To choose the activity each individual participates in at a given time step, we follow the same methodology as used in JUNE [13]. Each individual is assigned a Poisson parameter according to their age and sex, λ, and the probability that an individual does a certain activity during a given time step is determined through a Poisson process. Currently, Poisson parameters are determined only based on an individual's age and sex, however this can be adapted to other demographic and behaviour variables if data is available.

To assign activities to individuals, we first check if the individual does any activity at the given time step:

$$p(\text{ any activity } | \text{ age}, \text{sex }) = 1 - p(\text{ no activity } | \text{ age}, \text{sex}) \tag{1}$$

$$= 1 - \exp\left(-\sum_{a=1}^{N} \lambda_a(\text{age, sex})\ \Delta t\right), \tag{2}$$

where λ_a(age,sex) is the Poisson parameter associated with activity a for a person with a given age and sex, N is the number of possible activities and Δt is the amount of time allowed to do a given activity (currently this parameter is fixed at 2 hours,

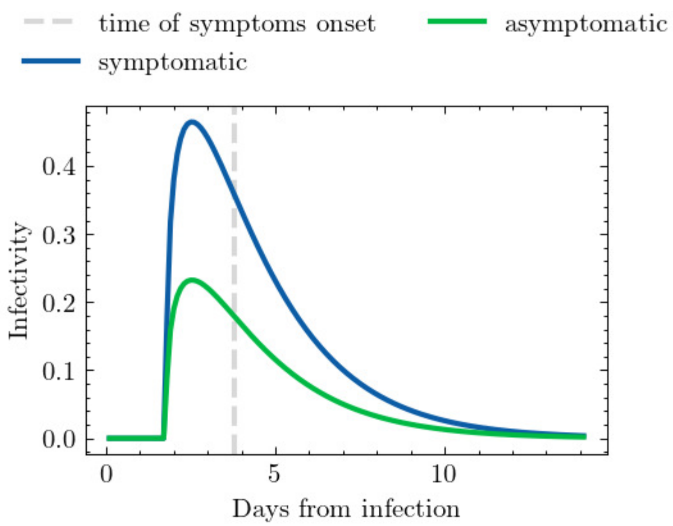

Fig 3. Infectiousness profile as a function of time derived from [14].

however can be varied if necessary). If no activity is performed then the individual returns to their shelter.

If a person carries out an activity, the next step is to determine which specific activity is chosen. The probability that activity a is chosen given that the person does any activity is given by:

$$p_a \quad = \quad \frac{\lambda_a(\text{age, sex})}{\sum_{j=1}^{N} \; \lambda_j(\text{age, sex})}, \tag{3}$$

Once an activity has been determined for a given individual, they are moved to the relevant location where they can interact with others who are also present.

When choosing which location of a given activity to attend, e.g. which communal center to attend, individuals in the model are given a choice of their nearest 5 locations corresponding to the relevant activity. This choice of nearest locations is decided heuristically and can be set by location-type as required if data is available to constrain this. We find that such a procedure gives rise to both local and inter-camp mixing.

Transmission

At each time step, different collections of individuals will inhabit the same space (e.g., a distribution center or play group), which we will refer to as a 'group'. Each group is a collection of individuals in the same geographical location performing the same activity. Individuals in the same group have the opportunity to interact with others in the group. If one or more of the people in the group are infected, there is a chance that a susceptible individual may become infected. To simulate the transmission of COVID-19, we implement the transmission dynamics described in Aylett-Bullock *et al.* [13], which we briefly outline here for completeness.

For a susceptible individual, s, we define the following properties: L is the location type (e.g., distribution center); g is the set of people present in a specific location; $i \in g$ is the subset of infectious people in that location; t is the current time step; Δt is the length of time that individuals remain in the location; ψ_s is the susceptibility of individual s; $\mathcal{I}_i(\cdot)$ is the infectiousness of individual i over the time frame t to $t + \Delta t$; $\beta_{si}^{(L,g)}(\cdot)$ characterizes the intensity of contacts between individuals s and i at a point in

time; and $\mathcal{P}_s(\cdot)$ is the probability that susceptible individual s is infected over a given period of time.

The probability of a person, s, being infected in the time step $[t, t+\Delta t]$ is taken to be:

$$\bar{\mathcal{P}}_s(t,\, t+\Delta t) = 1 - \exp\left[-\psi_s \sum_{i\in g} \int_t^{t+\Delta t} \beta_{si}^{(L,g)}(t')\mathcal{I}_i(t')\mathrm{d}t'\right], \tag{4}$$

As expected, staying longer in a location, being more susceptible to the disease, being surrounded by more infectious people, and having more intense contacts all increase the probability of disease transmission.

The intensity of the contacts, β, depends on the location, the number of people in the group, time, and the number of contacts along with the proportion of which are physical (as opposed to less intense conversational contacts). To represent this, the intensity of contacts between susceptible individual s and infectious individual i who are part of group g in location L at time t' is taken to be:

$$\beta_{si}^{(L,g)}(t') = \frac{\beta^{(L)}(t')}{|g|} \left\{\chi_{si}^{(L)}(t')\left[1 + \phi_{si}^{(L)}(t') \cdot \Big(\alpha(t') - 1\Big)\right]\right\}, \tag{5}$$

where $|g|$ is the number of people in group g, $\chi_{si}^{(L)}(t')$ is the number of contacts between people with the ages of individuals s and i over a given time period, $\phi_{si}^{(L)}(t')$ is the proportion of those contacts that are physical, and $\alpha(t')$ describes the additional infection risk of the physical contacts relative to the conversational ones.

In an ideal situation, the mixing matrices $\chi_{si}^{(L)}(t')$ would be derived from survey data [15–17], but such data has not been collected in the settlement setting. Instead we use a mixture of survey and observational data, along with various assumptions to hypothesize mixing matrices such that they capture the broad structure of contacts in a given location while allowing the values of the $\beta^{(L)}(t')$ parameters to absorb the details of the precise number of contacts. A similar argument can be made for the values of $\phi_{si}^{(L)}(t')$. Since $\alpha(t')$ is fixed for all locations, this just serves as a scaling parameter for the physical interaction matrix and so can be arbitrarily set. We heuristically take $\alpha(t') = 4$ to account for increased intensity likely found in the settlement environment. (The $\alpha(t')$ parameter serves an important purpose when fitting parameters to historical data, although this is not the focus of this work.) We detail our choices for $\chi_{si}^{(L)}(t')$ and relevant assumptions in the next section.

For modeling the spread of COVID-19, we use the time-dependent infectiousness profile, $\mathcal{I}_i(t')$, presented in [14] and represented in Fig 3. As in [13], we reduce the infectiousness of asymptomatic individuals, relative to that of a symptomatic individual, by a factor of 0.5.

When adapting the model to a disease other than COVID-19, one would need to modify the time-dependent infectiousness profile, and the intensity parameters that control the probability of transmission at different locations. Moreover, the different symptom related rates described in Section **??** would need to be adjusted to the observed outcomes of the new disease.

Mixing Matrices

Contact matrices are used by many modeling approaches, including ABMs, to specify the number of contacts between different people. Common sources of these matrices are surveys such as [15–17] from which contact matrices can be derived stratified by age

and type of location in which the contact was made. Many of these surveys also delineate between physical and conversational contacts, the former being more intense than the latter. We account for these different types of contact through specifying a physical contact multiplier which represents the proportion of all contacts in a given setting which are physical in nature.

While many surveys exist at the national level, it is not clear how these apply in the settlement setting in which living conditions, and cultural norms, can vary greatly from their host country, or of the PoC's country of origin. However, a key advantage of the June framework [13] is that the precise contact matrices do not need to be fully known in order to reproduce the age and location dependent mixing patterns found in reality. This is because, in reality, contact matrices are largely a function of an individual's movement patterns and the activities they participate in. Since we naturally incorporate this information in the setting of age and sex dependent probabilities that an individual goes to a certain location, we just need to focus on getting the broad character of the relative number of contacts correct, e.g. encouraging school children to be more likely to interact with each other than the teacher in a learning center. Furthermore, given the form of Equation 5, the scaling of the $\beta^{(L)}(t)$ parameter by location L absorbs the uncertainty of the total number of contacts in each location. A similar logic applies to the proportion of contacts which are physical in nature. A full discussion of this can be found in Section 4 and Appendix B of [13].

Adopting the naming convention of [13], we will call the input to our model "social mixing matrices" and the derived output the "contact matrices". Here we discuss the input social mixing matrices for each location which are designed to capture the broad character of the interactions in these venues. Given the degree to which full contact matrices are unknown in this setting, we aim to keep the input social mixing matrices simple so as to capture reasonable mixing but not overly bias results.

The choices of values in this section are chosen such that all matrices have a similar order of magnitude effect with the exception of learning centers, where the asymmetry in contacts between members of the participant groups (teachers and students) is most pronounced. This means that when we group indoor and outdoor intensity parameters ($\beta^{(L)}$ parameters) together for scenario modelling, we can create new scenarios easily by altering the relative values of these parameters without having to separately account for the multiplicative effect of the social mixing matrices. Furthermore, since this paper focuses on modeling the relative efficacies of interventions, some uncertainty in the relative social mixing matrix values is further divided.

Shelters

Some shelters in the Cox's Bazar settlement, and other settlements, are shared between up to two families. We assume that within each family, all individuals are equally likely to interact with each other given the proximity of the setting. Since some shared shelters contain dividing walls between families, but some do not, if a family shares a shelter with another family then we assume that each family is approximately half as likely to interact with the other family than within themselves. For the proportion of physical contacts, we assume this scaling to be a factor of 4 different with 80% of contacts within a household being physical, but only 20% of those with another household who shares the same shelter. This results in:

$$\mathcal{M}_{ij}^{(S)} = \begin{pmatrix} 5 & 2 \\ 2 & 5 \end{pmatrix} \text{ and } \phi_{ij}^{(S)} = \begin{pmatrix} 0.8 & 0.2 \\ 0.2 & 0.8 \end{pmatrix}, \quad (6)$$

where $\{i, j\} \in \{H, O\}$ for the household and other household entries respectively. Here, 5 is chosen as the intra-household contact baseline in order to ensure all social

mixing matrices have a similar order of magnitude effect on the probability of transmission.

Learning Centers

Learning centers have two key groups which can interact, students, S, and teachers, T. To ensure that students and teachers make proportionately the correct number of contacts given the average student-teacher ratio of 30:1 we set:

$$\mathcal{M}_{ij}^{(L)} = \begin{pmatrix} 5 & 15 \\ 0.5 & 5 \end{pmatrix}, \tag{7}$$

where $\{i, j\} \in \{T, S\}$ which is in close agreement with that derived in Appendix B.2 in [13].

We assume the proportion of physical interactions to be the same among all participants in the learning center given the closeness of the classroom setting and so this is absorbed in the intensity parameter value.

Play groups

When modelling interaction in play groups, for simplicity we assume children in the same age bracket play and interact with each other and do not interact with others outside their age bracket. We define age brackets to be: 3-6 year olds, $P1$, 7-11 year olds, $P2$, and 12-16 year olds, $P3$. The proportion of physical interactions are assumed to scale by age bracket:

$$\mathcal{M}_{ij}^{(PG)} = \begin{pmatrix} 5 & 0 & 0 \\ 0 & 5 & 0 \\ 0 & 0 & 5 \end{pmatrix} \text{ and } \phi_{ij}^{(PG)} = \begin{pmatrix} 0.6 & 0 & 0 \\ 0 & 0.3 & 0 \\ 0 & 0 & 0.15 \end{pmatrix}, \tag{8}$$

where $\{i, j\} \in \{P1, P2, P3\}$.

Other locations

Given the lack of available data on contact patterns, in all other locations in which interactions can occur, e.g. distribution centers and communal centers, we assume the social mixing and physical contact matrices to be single valued at $\mathcal{M}_{ij}^{(O)} = 5$ and $\phi_{ij}^{(O)} = 0.12$ respectively. This follows the same logic as that in Appendix B.4 of [13].

Comorbidities

Relative to previous simulations in refugee settings, one of our primary contributions is the inclusion of population-specific comorbidities in our model of COVID-19 risk. Accounting for such comorbidities is important because much of the data on COVID-19 severity comes from the developed world, where the prevalence of chronic illnesses such as diabetes or cardiovascular disease may be unusually high, and the prevalence of infectious diseases such as HIV/AIDS and tuberculosis may be relatively low. When using developed-country estimates of COVID-19 risk to estimate risk in the developing world, the differing prevalence of chronic illnesses may lead to an upward bias in the estimated risk of severe illness due to COVID-19, whereas the differing prevalence of infectious diseases may lead to a downward bias in the estimated risk of severe illness due to COVID-19. Therefore, properly estimating location-specific severity rates requires accounting for these differences in comorbidity distributions.

Our approach to accounting for comorbidities is based on the work of Clark *et al.* [18], who provide estimates of how 12 different types of comorbidities influence the

relative risk of developing severe disease as defined by "severe acute respiratory illness (fever and at least one sign/symptom of respiratory disease, e.g. cough, shortness of breath; AND requiring hospitalization)". In our model, we modify the definition of severe illness to allow for the possibility of severe cases and death without hospitalization. The complete list of conditions and risk multipliers which we adopted from Clark *et al.* is included in Table 1. Given detailed estimates of the risk of developing severe COVID-19 by age and sex in one country, Clark *et al.* propose a risk adjustment method to modify these estimates for the comorbidity mix of another country, which we adapt for our purposes and describe in more detail below.

Risk Group	Risk Score
HIV/AIDS	1.5
Tuberculosis	1.5
Cancers with direct immunosuppression	1.5
Cancers with possible immunosuppression	1.5
Cardiovascular diseases	3
Chronic respiratory diseases	1.5
Diabetes mellitus	3
Chronic kidney diseases	3
Chronic neurological disorders	1.5
Sickle cell disorders	1.5

Table 1. Comorbidity multipliers taken from Clark *et al.* [19]. These multipliers represent the relative risk of developing severe COVID-19, conditional on being affected by the comorbidity in question. A healthy individual has a relative risk of 1, implying that e.g. an HIV-positive person has a 1.5-times greater risk of developing severe COVID-19 than a healthy person.

We begin with data on the average risk of developing severe COVID-19 conditional on infection by age and sex cohort in the UK; this is the setting in which the JUNE model was developed, and was therefore an already-available source of detailed COVID-19 data. Presumably, these risk estimates already take into account the comorbidities which affect the relevant cohort in the UK. Therefore, we divide these average risk estimates by an adjustment factor in order to 'purge' the UK statistic of specific attributes relevant only to the UK population. The result is effectively a comorbidity-free probability of developing severe COVID-19 conditional on age and sex.

Concretely, we calculate the comorbidity-free age- and sex–specific risk of infection as:

$$P(\text{severe}\,|\,\text{age, sex}) = \frac{P^{UK}(\text{severe}\,|\,\text{age, sex})}{\bar{\lambda}^{UK}(\text{age, sex})} \tag{9}$$

where $P^{UK}(\text{severe}\,|\,\text{age, sex})$ is the probability of developing severe COVID-19 for a given age and sex cohort in the UK as described above and $\bar{\lambda}^{UK}(\text{age, sex})$ is the UK-specific adjustment factor. The latter is defined as:

$$\bar{\lambda}^{UK}(\text{age, sex}) = \frac{N^{UK}(\tilde{c}, \text{age, sex})}{N^{UK}(\text{age, sex})} + \frac{N^{UK}(\text{age, sex}) - N^{UK}(\tilde{c}, \text{age, sex})}{\sum_c N(c, \text{age, sex})} \left(\sum_c \lambda_c \frac{N^{UK}(c, \text{age, sex})}{N^{UK}(\text{age, sex})} \right) \tag{10}$$

where $\tilde{c}$ denotes individuals with no comorbidities; c indexes comorbidities; λ_c is the corresponding comorbidity multiplier from Table 1; $N^{UK}(c, \text{age, sex})$ represents the number of people in a given age and sex cohort that are afflicted by comorbidity c in the UK; and $N^{UK}(\text{age, sex})$ represents the total number of people in the age and sex cohort in the UK. The term in parentheses in Equation 10 is a weighted sum of

comorbidity multipliers, where the weights represent the fraction of the population with each comorbidity in the UK. The prefactor that multiplies the term in parentheses is a normalization term to ensure that the population fractions across all comorbidity conditions and the no-comorbidity condition sum to 1. This is necessary because a single individual may have multiple comorbidities, so the sum of empirically-observed population fractions over all comorbidity conditions could be greater than one.

Since comorbidity prevalence rates are provided for each comorbidity separately, we do not have data on how often particular comorbidities coincide. Therefore, we do not know how many people have no comorbidities ($\tilde{c}$). To calculate this number we follow the general strategy outlined in Clark *et al.* [18]. First, we assume that comorbidities are independently distributed, and calculate the expected proportion of people with one or more comorbidities; we then scale this proportion by 0.9 to account for likely correlations between comorbidities. Next, we confirm that the estimated proportion of people with one or more comorbidities is greater than the proportion of people with each single comorbidity; otherwise, we set the estimated proportion of people with one or more comorbidities equal to the proportion of people with the most common comorbidity. Subtracting this number from 1, we obtain the estimated proportion of people with no comorbidities. That is,

$$\frac{N^{UK}(\tilde{c},\,\text{age, sex})}{N^{UK}(\text{age, sex})} = 1 - \max\left[0.9\left(1 - \prod_{c}\left(1 - \frac{N^{UK}(c,\,\text{age, sex})}{N^{UK}(\text{age, sex})}\right)\right) \quad , \quad \max_{c}\left(\frac{N^{UK}(c,\,\text{age, sex})}{N^{UK}(\text{age, sex})}\right)\right]$$

We next attach a calculated age-, sex- and comorbidity-specific risk of severe infection to each agent in the model. The distribution of age and sex in the population is derived from settlement statistics, whereas the distribution of comorbidities is drawn from the age- and sex-specific comorbidity prevalence in Myanmar as estimated by the 2017 Global Burden of Disease (GBD) study and tabulated in Clark *et al.* [20, 21]. While there are active efforts to track illness in the camps, these efforts are oriented towards communicable disease and did not provide sufficient data to estimate the prevalence of the diseases in Table 1; therefore, we have used the population of Myanmar as the reference population for comorbidity prevalence estimates. We note that a national level characterization could be biased, however, there is currently a lack of available data at the relevant demographic scale. People living in remote locations with various predisposing risk factors may not be able to be generalized at the national level and vice versa. The use of comorbidity data is only used in our model to adjust the likelihood of disease progression, and therefore the chance that someone may be hospitalised. Since we take the approach of assessing the relative efficacies of operational interventions, these uncertainties will often be sub-dominant, however, in the event of any precise predictions these uncertainties should be clearly stated.

For each sampled individual, we calculate the risk of severe disease by adjusting the comorbidity-free risk estimate by the multiplier from Table 1 that is specific to the individual's assigned comorbidity:

$$P(\text{severe} \,|\, c,\,\text{age, sex}) \quad = \quad \lambda_c \; P(\text{severe} \,|\, \text{age, sex}) \tag{11}$$

This probability of severe infection can then be used to simulate the trajectory of each individual's illness, if infected.

Data Visualisation Tool

Our dashboard is built independently of the main simulation code and relies on a detailed data structure collected from an experiment (batch of simulations). Many things can happen to an agent in a single simulation. For example, at any time point an

agent can get infected, change locations, infect another, become hospitalized, die, or recover. Each of these 'events' can be reduced into a useful statistic (e.g., the number of infected each day or the locations where each agent got infected per day) and stored as a column of a CSV that is eventually consumed by the dashboard along with a metadata file describing the hyperparameters of each run. The dashboard is built with VueJS. All logic for processing the CSV files and displaying them to the user for interaction lives within the Typescript code. Snippets of primary dashboard functionality are shown in Figs 4, 5.

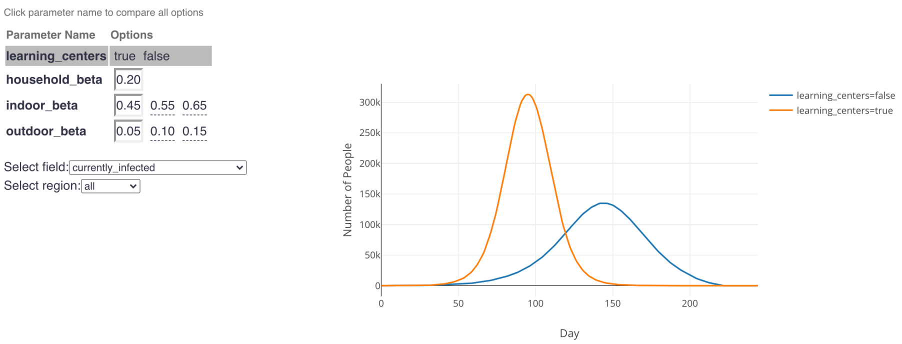

Fig 4. An example from the dashboard showing the infection curves (right) when learning centers are open (orange curve) or closed (blue curve) assuming specified interaction intensities. This graphic answers the question: "How can we expect infection rates to evolve if we open learning centers, assuming people follow mask and social distancing rules such that the indoor infection rate ("indoor_beta") and outdoor infection rate ("outdoor_beta") are low?" Different simulations with different interaction intensities can be selected by clicking different cells in the parameter grid (left). Select the name of a parameter (e.g., "learning_centers", "indoor_beta") to compare the selected field across all different values of that parameter while the other parameters remain constant. Note that the "beta" values are defined as in Equation 5 and all values are relative to the "household_beta" in this paper (see **??**).

The many facets presented by the dashboard assist the discovery of rich insights from the information-dense simulations. In addition to several of the plots shown throughout Section **??**, the dashboard allows users to zoom in to simulation results across different parameters for specifics (Fig 5) and zoom out for overviews and easier exploration (Fig 4). It can also show how different interventions vary by geography. How do the initial locations of infections impact the eventual geographical distribution of infections or deaths? How does medical infrastructure play a role in hospitalisation trends? The dashboard plots these trends on top of a regional choropleth map for every simulation and an interactively selected time step.

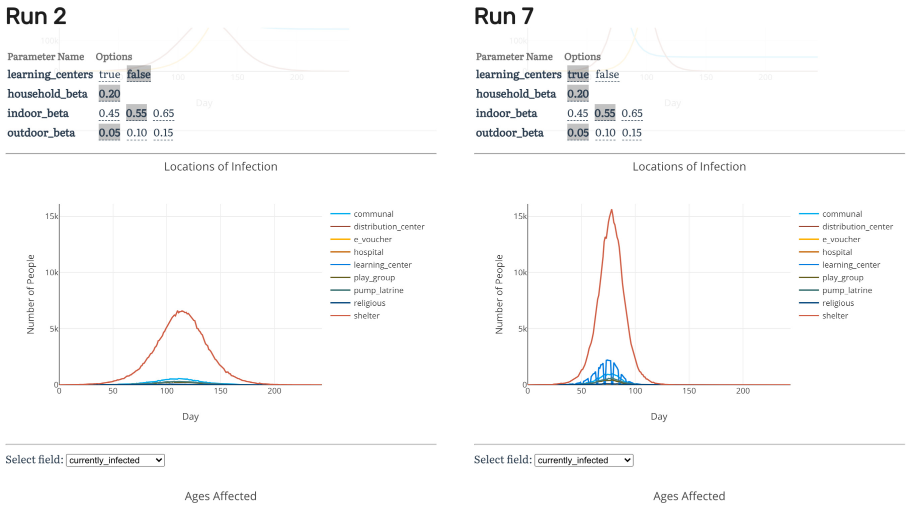

Fig 5. Direct comparison of two different simulations with learning centers open and closed. Displayed plots show distribution of where infections occurred each day (note that infections due to learning centers occur only on weekdays when learning centers are open). Scrolling up or down on this view shows additional comparison plots such as the SIR curve, ages affected, and geographical spread. Select a different run for each column by clicking around the parameter grid (top).

References

1. ISCG. Outline of camps of Rohingya refugees in Cox's Bazar;. `https://data.humdata.org/dataset/outline-of-camps-sites-of-rohingya-refugees-in-cox-s-bazar-bangladesh`.

2. IOM. IOM Bangladesh - Needs and Population Monitoring (NPM) Majhee Blocks Mapping;. `https://data.humdata.org/dataset/iom-bangladesh-needs-and-population-monitoring-npm-majhee-blocks-mapping`.

3. IOM. Needs and Population Monitoring (NPM) Round 16 Site Assessment; 2019. data retrieved from, `https://data.humdata.org/dataset/iom-bangladesh-needs-and-population-monitoring-npm-round-16-site-assessment`.

4. of Bangladesh UG. Block-level Population Fact Sheet; 2020. data retrieved from, `https://data2.unhcr.org/en/documents/details/82872`.

5. Joint Government of Bangladesh U. Population Factsheet; 2020. retrieved from, `https://data2.unhcr.org/en/documents/details/76920`.

6. Sector CE. Cox's Bazar - Gap Analysis for the Education Sector; 2020. data retrieved from, `https://drive.google.com/drive/folders/1B3kwTlKn_he_DiEKLtFa1C02toe7ApGW`.

7. Oxfam. Rohingya Refugee Response Gender Analysis; 2018. data retrieved from, `https://oxfamilibrary.openrepository.com/bitstream/handle/10546/620528/rr-rohingya-refugee-response-gender-analysis-010818-en.pdf?sequence=1`.

8. GAGE Y IPA. The lives they lead: Exploring the capabilities of Bangladeshi and Rohingya adolescents in Cox's Bazar, Bangladesh; 2019. retrieved from,

https://www.gage.odi.org/wp-content/uploads/2019/12/GAGE-Rohingya-brief_WEB-1.pdf.

9. GAGE Y IPA. ‘I don’t have any aspiration because I couldn’t study’: Exploring the educational barriers facing adolescents in Cox’s Bazar; 2020. retrieved from, https://www.gage.odi.org/publication/exploring-educational-barriers-facing-adolescents-coxs-bazar/.

10. ISCG. Bangladesh - Outline of camps of Rohingya refugees in Cox’s Bazar; 2021. data retrieved from, https://data.humdata.org/dataset/outline-of-camps-sites-of-rohingya-refugees-in-cox-s-bazar-bangladesh.

11. ISCG. Cox’s Bazar - Common facility mapping dataset; 2020. data retrieved from, https://data.humdata.org/dataset/cox-s-bazar-common-facility-mapping-dataset.

12. UNHCR. Rohingya Refugee Emergency at a Glance; 2018. retrieved from, https://unhcr.maps.arcgis.com/apps/Cascade/index.html?appid=5fdca0f47f1a46498002f39894fcd26f.

13. Aylett-Bullock J, Cuesta-Lazaro C, Quera-Bofarull A, Icaza-Lizaola M, Sedgewick A, Truong H, et al. JUNE: open-source individual-based epidemiology simulation. R. Soc. Open Sci. 2020;8: 210506. doi:10.1098/rsos.210506.

14. He X, Lau EH, Wu P, Deng X, Wang J, Hao X, et al. Temporal dynamics in viral shedding and transmissibility of COVID-19. Nature medicine. 2020;26(5):672–675.

15. Mossong J, Hens N, Jit M, Beutels P, Auranen K, Mikolajczyk R, et al. Social contacts and mixing patterns relevant to the spread of infectious diseases. PLoS Med. 2008;5(3):e74.

16. Prem K, Cook AR, Jit M. Projecting social contact matrices in 152 countries using contact surveys and demographic data. PLOS Computational Biology. 2017;13(9):1–21. doi:10.1371/journal.pcbi.1005697.

17. Klepac P, Kucharski AJ, Conlan AJ, Kissler S, Tang M, Fry H, et al. Contacts in context: large-scale setting-specific social mixing matrices from the BBC Pandemic project. medRxiv. 2020;.

18. Clark A, Jit M, Warren-Gash C, Guthrie B, Wang HH, Mercer SW, et al. Global, Regional, and National Estimates of the Population at Increased Risk of Severe COVID-19 Due to Underlying Health Conditions in 2020: A Modelling Study. The Lancet Global Health. 2020;doi:10.1016/S2214-109X(20)30264-3.

19. Clark A, Jit M, Warren-Gash C, Guthrie B, Wang HHX, Mercer SW, et al. Supplement to: Global, Regional, and National Estimates of the Population at Increased Risk of Severe COVID-19 Due to Underlying Health Conditions in 2020: A Modelling Study. The Lancet Global Health. 2020;.

20. GBD. Global Burden of Disease, Results Tool; 2017. data retrieved from, http://ghdx.healthdata.org/gbd-results-tool.

21. Clark A, Jit M, Warren-Gash C, Guthrie B, Wang HH, Mercer SW, et al.. Spreadsheet Tool for: Global, Regional, and National Estimates of the Population at Increased Risk of Severe COVID-19 Due to Underlying Health Conditions in 2020: A Modelling Study; 2020. https://cmmid.github.io/topics/covid19/Global_risk_factors.html.
