# Supplementary material for: Operational response simulation tool for epidemics within refugee and IDP settlements: A scenario-based case study of the Cox’s Bazar settlement
Source: PLoS Comput Biol. 2021 Oct 28;17(10):e1009360. doi: 10.1371/journal.pcbi.1009360 (PMC8553081; doi:10.1371/journal.pcbi.1009360)
Supplement: S2 Appendix — Parameters, and their accompanying sources, used in the constructing the digital twin and simulation. (PDF) [file pcbi.1009360.s002.pdf]

S2 Appendix

Digital twin and simulation parameters

In this section we detail the data sources for both the digital twin and simulation parameter ranges. By specifying the parameter choices made in the context of the Cox's Bazar settlement, we hope to enable simple adaptation of this approach to other settings, given appropriate available data. Note that in the case of the Cox's Bazar settlement, we had access to certain datasets which may not be available in other settings. In addition, different data may be required depending on the applications of the model.

Digital twin

Table 1 specifies the data sources for parameters relating to specific inputs for the creation of the digital twin of the Cox's Bazar settlement. These inputs include the locations of different infrastructure and facilities; population demographics and household composition; and access to health care as well as data on comorbidities. We bold-type the datasets which are necessary to adapt this model to other settings.

Description	Source
**Population Demographics and Geography**	UNHCR & Government of Bangladesh [1], IOM [2]
**Household Composition**	UNHCR & Government of Bangladesh [1], UNHCR [3]
Access to Healthcare	UNHCR [4]
Pump & Latrine Locations	UNHCR [3]
**Infrastructure Locations**	ISCG [5]
School Attendance & Children's Activities	Cox's Bazar Education Sector [6], Oxfam [7], GAGE/IPA/YALE [8,9]
Additional Hospital Locations	ISCG [10], Truelove et al. [11]
**Comorbidity Data**	Clark et al. [12], Global Burden of Disease study [13], UN Population Estimates [14]

Table 1. Data sources for the digital twin. Data sources in bold-type are those which are necessary to adapt the model to other settings.

Simulation

Table 2 specifies the data sources for parameters relating to specific inputs used in the creation of the simulator, which models the movement of people in the Cox's Bazar settlement. These inputs include information on the frequency of visits to facilities such as markets, distribution centers, and religious centers, dis-aggregated by age and sex. When adapting this model to other settings, data on how often individuals attend different locations is required. In the case of the Cox's Bazar settlement, these were derived from the reports interviews mentioned in Table 2, however, different data sources and assumptions may be required when adapting the model to other settings.

Description	Source
Distribution Centers	Hoddinott [15], Oxfam [7], ISCG & Government of Bangladesh [16,17], UNHCR [18], WFP [19,20]
Non-Food Distribution Centers	ISCG [5]
E-Voucher Outlets	WFP [19–21], Hoddinott [15]
Communal Centers (kitchens, centers, etc.)	ACAPS [22], Government of Bangladesh [23], WV [24], ISCG [5]
Female-friendly Spaces	UN Women [25], WFP [26]
Market Attendance	AAH [27]
Religious Center Attendance	Lopez et al. [28]
Learning Centers	HRW [29], Government of Bangladesh [30], Cox's Bazar Education Sector [6], ISCG [5], WFP [19]
Pump & Latrine Usage	UNHCR [3,31], WFP [19]

Table 2. Data sources for the simulator including literature relating to how often individuals attend different locations based on demographic characteristics.

Activities

In the previous section we detailed the data sources for both the digital twin of the Cox's Bazar settlement and the simulator to model the movement of people. In this section we discuss how these sources and others are used to derive assumptions which guide our choice of model inputs. Table 3 contains information regarding each of the possible activities which individuals in the model may perform at different time steps. We specify details of individual movements during normal operations (i.e. pre-COVID-19) and how these movements have changed since March 2020 when the settlement began introducing measures to mitigate the spread of COVID-19. Details explicitly included in our model are highlighted with an asterisk.

Table 3. Details and assumptions used in each activity included in the simulation. * indicates that the specific detail is currently being implemented in the simulation.

<table>
<tr><th>Activity</th><th>Normal Operation</th><th>During COVID-19</th><th>Sources</th></tr>
<tr><td>Distribution Centers</td><td>• As of 07/2020, 12% of PoCs receive their food rations from distribution centers*
• As of 09/2020, 6% of PoCs receive their food rations from distribution centers
• Households collect food (oil, rice, & pulses) twice a month*
• Attendance demographics are based on statistics taken from Fig 44 from [7], outlined below. Note, rates have been normalized after excluding missing values*
<table><tr><th></th><th><18</th><th>18+</th></tr><tr><td>Men</td><td>8%</td><td>68%</td></tr><tr><td>Women</td><td>23%</td><td>1%</td></tr></table></td><td>• Only one individual from each household can enter the distribution site*</td><td>[15–20]</td></tr>
<tr><td>Non-Food Distribution Centers</td><td>• Various distribution sites for shelter/non-food items are included as outlined in the ISCG Infrastructure data
• LPG is collected according to household size</td><td>• Currently closed with the exception of LPG distribution centers*
• Households collect food once a month and LPG once a month*
• Attendance demographics are based on statistics from the table in the Distribution Centers row above*</td><td>[5, 16, 18, 32]</td></tr>
</table>

Continued on next page

Table 3 – continued from previous page

Activity	Normal Operation	During COVID-19	Sources
E-Voucher Outlets	• As of 07/2020 88% of PoCs receive their food rations from e-voucher outlets*<br>• As of 09/2020 94% of PoCs receive their food rations from e-voucher outlets<br>• Twice monthly payment provided to a "debit card" possessed by the most senior female member of the household<br>• Attendance demographics are based on statistics from the table in the Distribution Centers row above*<br>• Host community members can sell fresh produce in e-voucher outlets	• Households collect a fixed basked of food once a month*<br>• Only one individual from each household can enter the e-voucher outlet*	[15, 19–21]
Communal Centers	• Community spaces open for various purposes (i.e. livelihood training or counselling) and included as found in the ISCG data set*	• The number of people attending communal spaces was reduced by about half for both sexes until mid April, after which men returned to normal attendance but attendance rates remained halved for women*	[5, 22, 23, 32]
Safe Spaces for Women and Girls	• Centers where women and girls can seek counselling, help, or participate in various activities as found in the ISCG data set*	• Currently closed*	[5, 25, 26, 32]
Religious Centers	• 77% of men older than 11 years old attend weekly religious gatherings*	• Attendance at religious meetings falls by approximately 66% until mid April, after which attendance returns to normal*	[28, 32]
Learning Centers	• Enrollment data is taken from Education Sector GAP Analysis*<br>• Class size obtained by dividing the number of enrolled students by the number of learning centers in each respective camp found in the ISCG data set*	• Closed with provision of some resources for home schooling*	[5, 6, 19, 23, 29, 30]

Continued on next page

Table 3 – continued from previous page

Activity	Normal Operation	During COVID-19	Sources
Play Groups	• Assume children are likely to meet up with others approximately twice a day for 2 hours each time*<br>• Assume play groups vary in size from 2-20 children*	• Children are more likely to play with other children during the day now that learning centers are closed*	[32]
Hand Pumps & Latrines	• As of 04/2020, there are 100 people per functioning hand pump and 35 people per functioning latrine<br>• As of 09/2020, there are 21 people per functioning latrine<br>• Assume an average of 68 people per hand pump or latrine combined*<br>• Hand pumps or latrines are distributed according to population at the sub-block level*	• No change*	[3, 19, 31]
Hospitals	• NGO clinics, government hospitals, and private clinics are available for PoCs to seek care<br>• There are 24 primary health care centers in the camp, with 910 additional beds available outside of the settlement for the Cox's Bazar region<br>• We include eight hospitals in our simulations, taken from the ISCG data set*	• Not all hospitals are available for COVID-19 patients, however, we do not claim to model the capacity of each individual hospital since doing so would involve a deeper understanding of health system decision making processes. We show only aggregated hospital data to avoid this extra source of uncertainty.*	[5, 33]

References

1. of Bangladesh UG. Block-level Population Fact Sheet; 2020. data retrieved from, https://data2.unhcr.org/en/documents/details/82872.

2. IOM. Needs and Population Monitoring (NPM) Round 16 Site Assessment; 2019. data retrieved from, https://data.humdata.org/dataset/iom-bangladesh-needs-and-population-monitoring-npm-round-16-site-assessment.

3. UNHCR. Rohingya Refugee Emergency at a Glance; 2018. retrieved from, https://unhcr.maps.arcgis.com/apps/Cascade/index.html?appid=5fdca0f47f1a46498002f39894fcd26f.

4. UNHCR. Multi Sector Needs Assessment: Cox's Bazar, Rohingya Refugee Response; 2019. data retrieved from, https://microdata.unhcr.org/index.php/catalog/208.

5. ISCG. Cox's Bazar - Common facility mapping dataset; 2020. data retrieved from, https://data.humdata.org/dataset/cox-s-bazar-common-facility-mapping-dataset.

6. Sector CE. Cox's Bazar - Gap Analysis for the Education Sector; 2020. data retrieved from, https://drive.google.com/drive/folders/1B3kwTlKn_he_DiEKLtFa1C02toe7ApGW.

7. Oxfam. Rohingya Refugee Response Gender Analysis; 2018. data retrieved from, https://oxfamilibrary.openrepository.com/bitstream/handle/10546/620528/rr-rohingya-refugee-response-gender-analysis-010818-en.pdf?sequence=1.

8. GAGE Y IPA. The lives they lead: Exploring the capabilities of Bangladeshi and Rohingya adolescents in Cox's Bazar, Bangladesh; 2019. retrieved from, https://www.gage.odi.org/wp-content/uploads/2019/12/GAGE-Rohingya-brief_WEB-1.pdf.

9. GAGE Y IPA. 'I don't have any aspiration because I couldn't study': Exploring the educational barriers facing adolescents in Cox's Bazar; 2020. retrieved from, https://www.gage.odi.org/publication/exploring-educational-barriers-facing-adolescents-coxs-bazar/.

10. ISCG. Cox's Bazar: Health facility registry; 2018. data retrieved from, https://data.humdata.org/dataset/health-facility-registry.

11. Truelove S, Abrahim O, Altare C, Lauer SA, Grantz KH, Azman AS, et al. The potential impact of COVID-19 in refugee camps in Bangladesh and beyond: A modeling study. PLOS Medicine. 2020;17(6):1–15. doi:10.1371/journal.pmed.1003144.

12. Clark A, Jit M, Warren-Gash C, Guthrie B, Wang HH, Mercer SW, et al. Global, Regional, and National Estimates of the Population at Increased Risk of Severe COVID-19 Due to Underlying Health Conditions in 2020: A Modelling Study. The Lancet Global Health. 2020;doi:10.1016/S2214-109X(20)30264-3.

13. GBD. Global Burden of Disease, Results Tool; 2017. data retrieved from, http://ghdx.healthdata.org/gbd-results-tool.

14. UN. UN 2020, Mid-Year, Population Estimates; 2019. data retrieved from, https://population.un.org/wpp/Download/Standard/Interpolated/.

15. Hoddinott J, Dorosh P, Filipski M, Rosenbach G, Tiburcio E. Food transfers, electronic food vouchers and child nutritional status among Rohingya children living in Bangladesh. PLOS ONE. 2020;15(4):1–15. doi:10.1371/journal.pone.0230457.
16. of Bangladesh IG. LPG Project: RRRC Requirements; 2018. data retrieved from, https://www.humanitarianresponse.info/sites/www.humanitarianresponse.info/files/documents/files/rrrc_requirements_for_lpg_projects_-_july_2018.pdf.
17. ISCG. Situation report Rohingya Refugee Crisis, Cox's Bazar — September 2020; 2020. retrieved from, https://reliefweb.int/sites/reliefweb.int/files/resources/final_iscg_sitrep_-_september_2020.pdf.
18. UNHCR. LPG Distribution Tracking Dashboard; 2020. retrieved from, https://reliefweb.int/sites/reliefweb.int/files/resources/76372.pdf.
19. WFP. WFP Bangladesh Rohingya Refugee Response Situation Report #40; 2020. retrieved from, https://reliefweb.int/sites/reliefweb.int/files/resources/CXB%20External%20Sitrep%20%2340%20%28July%202020%29.pdf.
20. WFP. Key messages –WFP food distributions June 2020; 2020. retrieved from, https://fscluster.org/sites/default/files/documents/key_messages_covid_19_gfa_operations_june_2020_final_-_english.pdf.
21. WFP. Bringing greater economic opportunities for the host community, WFP reopens farmers' markets in Rohingya camps; 2020. retrieved from, https://reliefweb.int/report/bangladesh/bringing-greater-economic-opportunities-host-community-wfp-reopens-farmers-markets.
22. ACAPS. Rohingya RESPONSE Impact of COVID-19 on gender programming; 2020. retrieved from, https://reliefweb.int/sites/reliefweb.int/files/resources/20200608_acaps_coxs_bazar_analysis_hub_rohingya_response_covid19_and_gender_0.pdf.
23. Government of the People's Republic of Bangladesh Office of the Refugee Relief and Repatriation Commissioner. Rohingya refugee camp operations: Restricted Programmes in light of COVID-19; 2020. retrieved from, http://rrrc.gov.bd/sites/default/files/files/rrrc.portal.gov.bd/notices/48cc8a3a_58ab_42cf_93dc_b87a70a3d87d/2020-05-10-11-10-97a8138de4e490d8fe88a6dc393379f7.pdf.
24. Vision W. Innovative solutions help refugees in need worldwide; 2019. retrieved from, https://www.worldvision.org/refugees-news-stories/innovative-solutions-help-refugees-worldwide.
25. Women U. Photo Essay: "I want to live in peace"; 2018. retrieved from, https://www.unwomen.org/en/digital-library/multimedia/2018/5/photo-rohingya-women-refugees.
26. WFP. Creating spaces to train & empower women in Cox's Bazar; 2019. retrieved from, https://insight.wfp.org/wfp-and-unfp-inaugurate-womens-centres-af09268a6bdb.
27. Hunger AA. RAPID MARKET ASSESSMENT IN Cox's Bazar; 2017. retrieved from, https://www.humanitarianresponse.info/sites/www.humanitarianresponse.info/files/assessments/rma_report_acf_coxs_sept._2017_0.pdf.

28. Lopez-Pena P, Davis CA, Mobarak AM, Raihan S. Prevalence of COVID-19 Symptoms, Risk Factors, and Health Behaviors in Host and Refugee Communities in Cox's Bazar: A Representative Panel Study. Bull World Health Organ. 2020;.

29. HRW. "Are We Not Human?" Denial of Education for Rohingya Refugee Children in Bangladesh; 2019. data retrieved from, `https://www.hrw.org/report/2019/12/03/are-we-not-human/denial-education-rohingya-refugee-children-bangladesh`.

30. Government of the People's Republic of Bangladesh Office of the Refugee Relief and Repatriation Commissioner. Rohingya refugee camp operations: Essential Programmes in light of COVID-19; 2020. retrieved from, `http://rrrc.gov.bd/sites/default/files/files/rrrc.portal.gov.bd/notices/c3aece34_0550_4b4d_b33c_e8864272ada9/2020-03-25-16-34-21d19f130456961e35a25dbd1e5ef780.pdf`.

31. UNHCR. UNHCR Bangladesh - Operational Dashboard: 2020 Indicators Monitoring (As of September 2020); 2020. retrieved from, `https://data2.unhcr.org/en/documents/details/82821`.

32. Conversations with Cox's Bazar refugee operation Displacement Operations Team; 2020.

33. Truelove S, Abrahim O, Altare C, Lauer SA, Grantz KH, Azman AS, et al. The potential impact of COVID-19 in refugee camps in Bangladesh and beyond: A modeling study. PLOS Medicine. 2020;17(6):1–15. doi:10.1371/journal.pmed.1003144.
