# Supplementary material for: Operational response simulation tool for epidemics within refugee and IDP settlements: A scenario-based case study of the Cox’s Bazar settlement
Source: PLoS Comput Biol. 2021 Oct 28;17(10):e1009360. doi: 10.1371/journal.pcbi.1009360 (PMC8553081; doi:10.1371/journal.pcbi.1009360)
Supplement: S3 Appendix — Description of the initial seeding and intensity parameter settings for simulation runs as well as details on the baseline model, and stochasticity. (PDF) [file pcbi.1009360.s003.pdf]

S3 Appendix

Seeding, intensity parameters, and the baseline model

Initial seeding

To initialize the infection we use data from the WHO EWARS datasets [1] on confirmed cases by camp on the 24th of May. By that time, only six of the camps had detected at least one case of COVID-19, with case numbers totaling 22 across the Expansion Site. To account for under-reporting and other factors we conservatively scale this number by a factor of four. To seed the infection, we assume 88 people are COVID-positive and infectious at the beginning of the simulation. Half of these case are assumed to be distributed in the six camps with confirmed cases weighted by their relative prevalence, and the other 44 cases are randomly distributed among the remaining camps relative to their population size.

Given this choice of seeding by location, our baseline model was found to reproduce the geographic spread of the virus in its early stages within reasonable agreement. However, use of data for comparison beyond this initial phase is challenging due to the limitations mentioned previously.

Intensity parameter selection

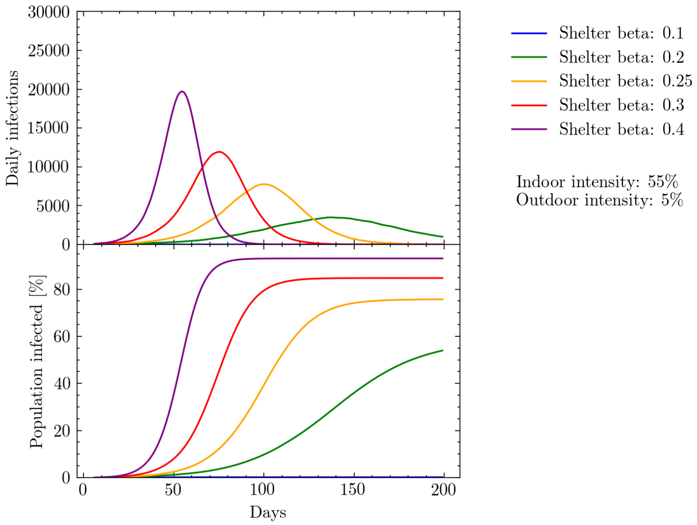

Fig 1. Simulated 7-day rolling average daily and cumulative infections measured in days since the beginning of the simulation. Results show the effects of varying the shelter intensity parameter while keeping the indoor and outdoor intensity parameters fixed at 55% and 5% respectively relative to the shelter intensity value.

The interaction intensity parameters ($\beta^{(L)}$ parameters - see Equation **??**) control multiplicatively the intensity of interactions in different locations L. In the event where

historical data is available to fit to, thereby enabling model predictions, these parameters would act as free fitting parameters [2]. However, in settings with limited available data on COVID-19 cases and other statistics, fitting a model to data is not possible. To account for this we focus on modeling the relative effects of different operational interventions, rather than precise statistics, and set these intensity parameters heuristically.

We group together intensity parameters into three categories: shelter, indoor and outdoor. Each location, and its corresponding intensity parameter is categorised into one of these groups following the location type given in Table **??**. While in reality each of the locations in a given group might have a different intensity parameter value, for simplicity we will assume the members of each group share the same intensity parameter value unless specified otherwise, i.e. people interact in all indoor locations with the same intensity with the exception of in the shelter environment, and people interact in all outdoor locations with the same intensity.

To set the parameters we first assume a hierarchy of values: shelter > indoor > outdoor. We then assume that the interaction intensity in indoor spaces outside the shelter environment is approximately half that of inside the shelter. This choice is designed to heuristically capture the fact that you will be less likely to interact with others outside your family in non-shelter settings as intensely as you would interact with your own family in the shelter. Given the density of shelters in the Cox's Bazar settlement, we feel this is a reasonable assumption. Although, there is little research on the subject, what studies have been carried out suggest that outdoor transmission is significantly less likely than indoor [3–5]. Indeed, reports making explicit calculations comparing the probability of transmission estimate that indoor transmission could be as much as 18-19 times more likely than outdoor [4]. Since we cannot give a definitive quantification of this intensity, we fix this parameter conservatively at 5% of the shelter interaction intensity parameter.

In some scenarios, since we are modeling the relative effects of interventions, the exact choices of these assumed values are less important. However, when modelling scenarios in which the specific interaction intensity ratios become more dominant, we vary the ratios of the intensity parameters to explicitly capture some of this uncertainty.

In all scenarios the value of the shelter intensity parameter is fixed and all other intensity parameters varied relative to this value. To arrive at a value for the shelter intensity we scanned several possible values while fixing the relative indoor and outdoor intensity values as stated above. Fig 1 shows the results of this parameter scan over a 200 day period since the beginning of the simulation. Early data from the WHO EWARS dataset [1] did not suggest a large initial surge of infections in the camp of the magnitude as might be expected by high value choices of the shelter intensity parameters. Therefore we heuristically choose a value of 0.25 since this scenario allows for different scenarios of disease transmission, both more and less severe than this baseline thereby enabling easy comparison. From the perspective of the timing of the peak, this scenario is in good agreement with the 'moderate transmission' scenario presented in [6] which is estimated to be equivalent to a basic reproduction number of $R_0 \approx 2.0 - 3.0$.

It should be noted that since the value of the shelter intensity parameter remains fixed in all scenarios, and no scenario modeled thus far has altered this intensity parameter, that the parameter simply acts as a scaling factor for the growth rate of the disease across all scenarios. Therefore, its relative effects, again, will largely divide out when comparing across scenarios making scenario-based modeling less sensitive to this parameter value. Furthermore, Fig 1 clearly demonstrates our model's ability to simulate a wide range of different transmission scenarios if required, along with its flexibility to fit to historical data.

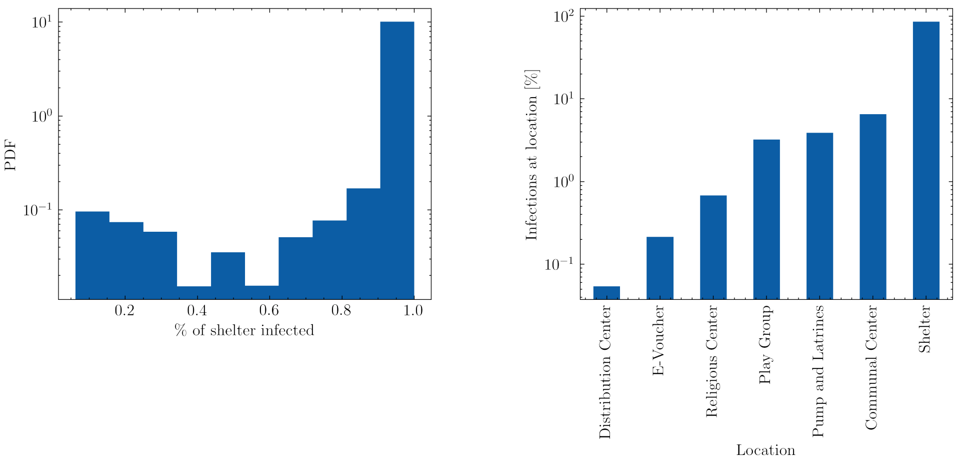

Fig 2. Left: the probability density function (PDF) of the percentage of individuals infected in a shelter, given that there has been at least one infection in the given shelter. Right: percentage of total infections that happened at each of the available locations by the end of the simulation.

In order to characterize the baseline and facilitate comparisons, in Fig 2 we show the percentage of infections occuring at each location by the end of the simulation for one realisation, and the probability of finding a certain percentage of a shelter infected given that there has been one infection in the shelter.

Stochasticity

In the previous section we set the parameters for the baseline model against which interventions are compared. As mentioned in Section **??**, the parameters chosen to be adjusted when assessing different intervention strategies are based on those which explore large regions of parameter space to characterise the effects of parameter uncertainty on possible scenarios. The other major contributor to uncertainties in epidemic models are the stochastic effects introduced through random probabilistic choices. In Fig 3, we present the results of rerunning the baseline model 50 times. Each simulation was run with a different random seed and a newly initiated 'digital twin' meaning households and other probabilistically determined entities are reinitialized in each run. Clearly, the model is relatively stable to stochastic effects, and these uncertainties are negligible when compared to the variations observed when exploring parameter space for different intervention strategies.

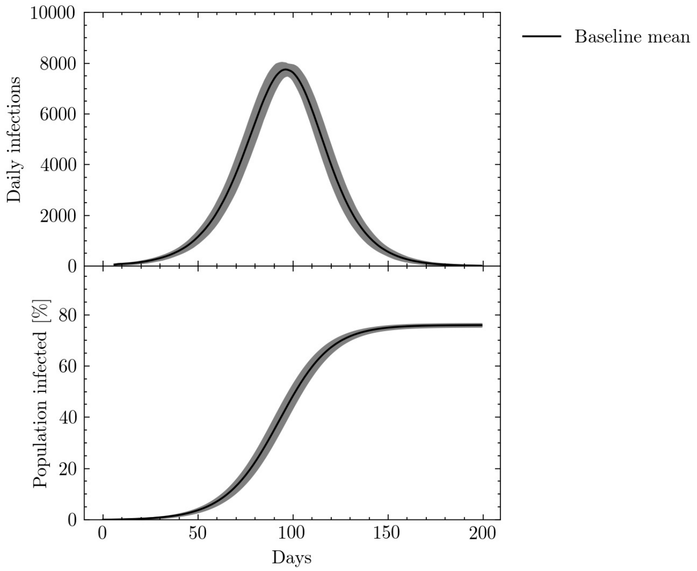

Fig 3. Mean baseline simulated 7-day rolling average daily and cumulative infections presented with two standard deviations over 50 reruns of the baseline model each with different random seed choices.

References

1. WHO. Bangladesh - Rohingya Crisis: Eearly Warning, Alert & Response System;. `https://www.who.int/bangladesh/emergencies/Rohingyacrisis/ewars`.

2. Aylett-Bullock J, Cuesta-Lazaro C, Quera-Bofarull A, Icaza-Lizaola M, Sedgewick A, Truong H, et al. JUNE: open-source individual-based epidemiology simulation. R. Soc. Open Sci. 2020;8: 210506. doi:10.1098/rsos.210506.

3. Qian H, Miao T, Li L, Zheng X, Luo D, Li Y. Indoor transmission of SARS-CoV-2. medRxiv. 2020;.

4. Nishiura H, Oshitani H, Kobayashi T, Saito T, Sunagawa T, Matsui T, et al. Closed environments facilitate secondary transmission of coronavirus disease 2019 (COVID-19). MedRxiv. 2020;.

5. Schuit M, Ratnesar-Shumate S, Yolitz J, Williams G, Weaver W, Green B, et al. Airborne SARS-CoV-2 is Rapidly Inactivated by Simulated Sunlight. The Journal of Infectious Diseases. 2020;.

6. Truelove S, Abrahim O, Altare C, Lauer SA, Grantz KH, Azman AS, et al. The potential impact of COVID-19 in refugee camps in Bangladesh and beyond: A modeling study. PLOS Medicine. 2020;17(6):1–15. doi:10.1371/journal.pmed.1003144.
