# Supplementary material for: Operational response simulation tool for epidemics within refugee and IDP settlements: A scenario-based case study of the Cox’s Bazar settlement
Source: PLoS Comput Biol. 2021 Oct 28;17(10):e1009360. doi: 10.1371/journal.pcbi.1009360 (PMC8553081; doi:10.1371/journal.pcbi.1009360)
Supplement: S1 Table — Additional details and discussions on exact parameter choices for certain operational interventions. (PDF) [file pcbi.1009360.s004.pdf]

S4 Tables

Intervention details

Parameter	Values
Symptomatic quarantine compliance	Fixed: 30%
Household quarantine compliance	Fixed: 20%
Testing compliance	Varied: 50%-100%
Average time delay between symptom onset and going to a treatment center	Varied: 5-10 days
Standard deviation of the time delay between symptom onset and going to a treatment center	Fixed: 1 day
Time spent in isolation inside a treatment center if tested positive	Varied: 5-10 days

Table 1. Isolation Center Simulation Parameters
The time delay between the onset of symptoms and going to a treatment center is drawn from a Normal distribution. In reality, tests used in the Cox's Bazar settlement take approximately 2 days to be processed and therefore choosing a mean time delay to isolation of 2 days presents a reasonable scenario. Including time delays as low as 0 days with high compliance rates presents a best-case scenario in which tests can be rapidly processed.

Intervention	Details [Compliance (%), Isolation time (days), Time delay to isolation (days)]	Cumulative Infections	Peak Intensity	Peak Timing
Baseline	No isolation	433298	7769	Day 100
1	[100, 10, 0]	434435	7911	Day 103
2	[100, 10, 1]	434194	7707	Day 99
3	[100, 10, 2]	431868	7727	Day 102
4	[100, 10, 3]	431854	7712	Day 104
5	[100, 10, 5]	433298	7858	Day 105
6	[100, 5, 2]	432042	7709	Day 96

Table 2. Isolation Centers: a summary of the isolation centers vs. home-care scenario simulations. Numbers are presented as 7-day rolling averages. Across all interventions, there is minimal change in the cumulative number of infections, peak intensity and peak timing. Note, that these data are for one simulation run only, however, the stochastic behaviour of the model is minimal.

Parameter	Value
Mask wearing compliance	Rate of people wearing masks correctly
Mask wearing efficacy	Estimated rate based on mask filtration ability, mask fit, and mask reuse [1].
Mask filtration ability	Depends on no. of layers/type of fabric
Mask wearing compliance rate	Varied: 10%-100%
Mask wearing efficacy rate	Varied: 10%-90%
Single layer cotton mask efficacy	Estimate: 50% [2–4]
Surgical mask efficacy	Estimate: 80% [2–4]

Table 3. Mask Wearing Simulation Parameters
The effect of mask wearing in different locations depends on mask efficacy and mask wearing compliance. For simplicity, the total compliance was varied equally across all locations. Note that literature on the efficacy of mask wearing is in preliminary stages and/or has small sample sizes, making it difficult to draw precise conclusions from the results.

Intervention	Details [Mask Efficacy (%), Mask Compliance (%)]	Cumulative Infections	Peak Intensity	Peak Timing
Baseline	No masks	434249	7925	Day 95
1	[20, 25]	422831	7327	Day 102
2	[20, 50]	406014	6292	Day 110
3	[20, 100]	367584	4739	Day 121
4	[50, 25]	400031	5961	Day 103
5	[50, 50]	340421	4233	Day 127
6	[50, 100]	61038	766	Day 199
7	[80, 25]	366205	4951	Day 119
8	[80, 50]	166827	2133	Day 193
9	[80, 100]	4281	69*	Day 9*

Table 4. Mask Wearing: a summary of the mask wearing simulations. Numbers are presented as 7-day rolling averages. Low cumulative infections, peak intensity and delayed peaks are observed for higher efficacy masks and compliance rates. For interventions marked with an *, these figures may be misleading as the statistics are so low for these runs that they can be considered random fluctuations. Note, that these data are for one simulation run only, however, the stochastic behaviour of the model is minimal.

Parameter	Definition/Value
Indoor setting/shelter interaction intensity	Varied: [45%, 55%, 65%]
Outdoor setting/shelter interaction intensity	Varied: [5%, 10%, 15%]

Table 5. Learning Center Reopening Simulation Parameters
Under the assumption of no mitigation strategies, opening learning centers depends on the choice of interaction intensity parameters. We vary each of the indoor and outdoor interaction intensities while keeping the other fixed.

Intervention	Details [Learning Center Status, Household Intensity (%), Outdoor Intensity (%), Indoor Intensity (%)]	Cumulative Infections	Peak Intensity	Peak Timing
1	[Open, 25%, 5%, 45%]	505749	14887	Day 65
2	[Open, 25%, 5%, 55%]	522908	18254	Day 57
3	[Open, 25%, 5%, 65%]	533816	20659	Day 54
4	[Closed, 25%, 5%, 45%]	395970	5929	Day 110
5	[Closed, 25%, 5%, 55%]	434594	8003	Day 89
6	[Closed, 25%, 5%, 65%]	461328	9568	Day 86
7	[Open, 25%, 15%, 55%]	554736	26376	Day 45
8	[Open, 25%, 10%, 55%]	544871	23117	Day 50
9	[Closed, 25%, 15%, 55%]	542801	19054	Day 59
10	[Closed, 25%, 10%, 55%]	514001	14287	Day 69

Table 6. **Learning Center:** a summary of the re-opening learning centers simulations. Numbers are presented as 7-day rolling averages. Since we vary both the indoor and outdoor intensities with learning centers open and closed we do not have a baseline run. We can see that in all comparable scenarios, opening the learning centers can increase the risk of infection. Note, that these data are for one simulation run only, however, the stochastic behaviour of the model is minimal.

Intervention	Values<br>(Interaction intensities are relative to the baseline indoor interaction intensity which is set at 55% of the shelter interaction intensity)	Details	Sources
Learning center attendance	Varied: [Daily, Every other day]	• Attendance every other day results in classes of half the size	
Extra learning centers	Varied: [10,50,100]	• Children from schools with the largest class sizes are sent to an extra learning center	
Learning center interaction intensity	Varied: [20%, 35%, 55%, 70%, 90%]	• Varied intensities can correspond to the interventions listed below	
Physical distancing interaction intensity	Estimated: 30%-50%	• Effectively means halving the class size<br>• Assumed less effective in practice (i.e. compared to mask wearing) as it is harder to strictly enforce in smaller rooms with a teacher moving around	[5–7]
Mask wearing interaction intensity	Estimated: 30%-50%	• Given constant supervision by teachers, can assume high likelihood of proper/constant wear by students<br>• Teachers would wear a mask unless they are speaking from the front of the classroom/cannot be understood by students while wearing a mask	[3–8]
Mask wearing & physical distancing interaction intensity	Estimated: 20%-30%	• Halving the class size<br>• Mask wearing enforced by teachers	[3–8]
Mask wearing, physical distancing, & ventilation interaction intensity	Estimated: 10%-20%	• Halving class size<br>• Mask wearing enforced by teachers<br>• Opening all windows, keeping doors open, and adding electric fans to increase air flow	[3–10]

Table 7. Mitigation strategies for Reopening Learning Centers Simulation Parameters

The existing literature provides some guidance on the effects that specific interventions within the classroom may have on the evolution of COVID-19 within the settlement. We consider a baseline case in which learning centers are open with no interventions. Note, with respect to ventilation, options in schools vary with the type of classroom, e.g. some learning centers are built from bamboo allowing for more natural air to flow, while others appear to be smaller, concrete rooms [11, 12]. In enclosed settings, ventilation could consist of opening windows and doors as well as using electric fans to increase air flow. Finally, we note that since the classrooms are relatively small, with a suggested size of 40 square meters and a recommended class size of 35-40 students, effective social distancing is not possible without reducing the class size through alternating attendance or opening new classrooms [13].

Intervention	Details [Attendance, Extra Learning Centers, Learning Center Intensity (%)]	Cumulative Infections	Peak Intensity	Peak Timing
Baseline	No Learning Centers	430621	7846	Day 94
Baseline	[Daily, 0, 100%]	522525	17832	Day 58
1	[Alternating, 0, 100%]	496855	13473	Day 71
2	[Daily, 10, 100%]	522525	17832	Day 58
3	[Daily, 50, 100%]	523620	18060	Day 58
4	[Daily, 100, 100%]	524145	18542	Day 56
5	[Daily, 0, 20%]	453976	9100	Day 86
6	[Daily, 0, 55%]	497084	13678	Day 67
7	[Daily, 0, 90%]	520011	17119	Day 59

Table 8. Learning Center Mitigations: a summary of the learning center re-opening mitigation learning centers simulations. Intensity is compared to the baseline learning center intensity (55% that of the shelter intensity). Numbers are presented as 7-day rolling averages. Alternating daily attendance of learning centers and introducing a range of intensity reducing measures (such as those in Table 7) can significantly improve all metrics. Adding extra learning centers is not observed to make a noticeable difference. Note, that these data are for one simulation run only, however, the stochastic behaviour of the model is minimal.

References

1. Toomey E, Conway Y, Burton C, Smith S, Smalle M, Chan XH, et al. Extended use or re-use of single-use surgical masks and filtering facepiece respirators: A rapid evidence review. medRxiv. 2020;.

2. Liang M, Gao L, Cheng C, Zhou Q, Uy JP, Heiner K, et al. Efficacy of face mask in preventing respiratory virus transmission: A systematic review and meta-analysis. Travel medicine and infectious disease. 2020;36:101751–101751.

3. Howard J, Huang A, Li Z, Tufekci Z, Zdimal V, van der Westhuizen HM, et al. Face masks against COVID-19: an evidence review. 2020;.

4. Fischer EP, Fischer MC, Grass D, Henrion I, Warren WS, Westman E. Low-cost measurement of face mask efficacy for filtering expelled droplets during speech. Science Advances. 2020;6(36):eabd3083.

5. Chu DK, Akl EA, Duda S, Solo K, Yaacoub S, Schünemann HJ, et al. Physical distancing, face masks, and eye protection to prevent person-to-person transmission of SARS-CoV-2 and COVID-19: a systematic review and meta-analysis. The Lancet. 2020;.

6. Wang Y, Tian H, Zhang L, Zhang M, Guo D, Wu W, et al. Reduction of secondary transmission of SARS-CoV-2 in households by face mask use, disinfection and social distancing: a cohort study in Beijing, China. BMJ Global Health. 2020;5(5):e002794.

7. Ahmed F, Zviedrite N, Uzicanin A. Effectiveness of workplace social distancing measures in reducing influenza transmission: a systematic review. BMC public health. 2018;18(1):518.

8. Feng Y, Marchal T, Sperry T, Yi H. Influence of wind and relative humidity on the social distancing effectiveness to prevent COVID-19 airborne transmission: A numerical study. Journal of aerosol science. 2020; p. 105585.

9. Atkinson J, Chartier Y, Pessoa-Silva CL, Jensen P, Li Y, Seto WH. Natural ventilation for infection control in health-care settings. World Health Organization; 2009.

10. Dai H, Zhao B. Association of infected probability of COVID-19 with ventilation rates in confined spaces: a Wells-Riley equation based investigation. medRxiv. 2020;.

11. Sector CE. Public Folder: BRAC, Two-Storey Learning Centers;. retrieved from, `https://drive.google.com/drive/folders/1WaIFGfe6k9jXz63QbAHx8jVRk9-2Ipw0`.

12. UK AI. Bangladesh: Estimated 500,000 Rohingya children being denied an education; 2019. retrieved from, `https://www.amnesty.org.uk/press-releases/bangladesh-estimated-500000-rohingya-children-being-denied-education`.

13. Sector CE. Guidance Note: Education Sector Standards for Rohingya Response;. retrieved from, `https://drive.google.com/drive/folders/1HqT3RmYUW8Z5B-q1gpqrYOo1SYaWJP_a`.
